# Multi-center retrospective cohort study applying deep learning to electrocardiograms to identify left heart valvular dysfunction

**DOI:** 10.1038/s43856-023-00240-w

**Published:** 2023-02-14

**Authors:** Akhil Vaid, Edgar Argulian, Stamatios Lerakis, Brett K. Beaulieu-Jones, Chayakrit Krittanawong, Eyal Klang, Joshua Lampert, Vivek Y. Reddy, Jagat Narula, Girish N. Nadkarni, Benjamin S. Glicksberg

**Affiliations:** 1 The Hasso Plattner Institute for Digital Health at Mount Sinai, Icahn School of Medicine at Mount Sinai, New York, NY USA; 2 Department of Genetics and Genomic Sciences, Icahn School of Medicine at Mount Sinai, New York, NY USA; 3 Mount Sinai Heart, Icahn School of Medicine at Mount Sinai, New York, NY USA; 4 Department of Cardiology, Mount Sinai Morningside Hospital, Icahn School of Medicine at Mount Sinai, New York, NY USA; 5 Department of Biomedical Informatics, Harvard Medical School, Boston, MA USA; 6 Section of Biomedical Data Science, Department of Medicine, University of Chicago, Chicago, IL USA; 7 Cardiology Division, NYU Langone Health and NYU School of Medicine, New York, NY USA; 8 Sheba Medical Center, Department of Diagnostic Imaging, Tel Hashomer, Israel; 9 Sackler Medical School, Tel Aviv University, Tel Aviv, 52621 Israel; 10 Helmsley Electrophysiology Center, Icahn School of Medicine at Mount Sinai, New York, NY USA; 11 The Zena and Michael A. Wiener Cardiovascular Institute, Icahn School of Medicine at Mount Sinai, New York, NY USA; 12 The Charles Bronfman Institute for Personalized Medicine, Icahn School of Medicine at Mount Sinai, New York, NY USA; 13 The Division of Data Driven and Digital Medicine (D3M), The Department of Medicine, Icahn School of Medicine at Mount Siniai, New York, NY USA

**Keywords:** Cardiology, Health care, Computational biology and bioinformatics, Cardiovascular biology

## Abstract

**Background:**

Aortic Stenosis and Mitral Regurgitation are common valvular conditions representing a hidden burden of disease within the population. The aim of this study was to develop and validate deep learning-based screening and diagnostic tools that can help guide clinical decision making.

**Methods:**

In this multi-center retrospective cohort study, we acquired Transthoracic Echocardiogram reports from five Mount Sinai hospitals within New York City representing a demographically diverse cohort of patients. We developed a Natural Language Processing pipeline to extract ground-truth labels about valvular status and paired these to Electrocardiograms (ECGs). We developed and externally validated deep learning models capable of detecting valvular disease, in addition to considering scenarios of clinical deployment.

**Results:**

We use 617,338 ECGs paired to transthoracic echocardiograms from 123,096 patients to develop a deep learning model for detection of Mitral Regurgitation. Area Under Receiver Operating Characteristic curve (AUROC) is 0.88 (95% CI:0.88–0.89) in internal testing, and 0.81 (95% CI:0.80–0.82) in external validation. To develop a model for detection of Aortic Stenosis, we use 617,338 Echo-ECG pairs for 128,628 patients. AUROC is 0.89 (95% CI: 0.88-0.89) in internal testing, going to 0.86 (95% CI: 0.85-0.87) in external validation. The model’s performance increases leading up to the time of the diagnostic echo, and it performs well in validation against requirement of Transcatheter Aortic Valve Replacement procedures.

**Conclusions:**

Deep learning based tools can increase the amount of information extracted from ubiquitous investigations such as the ECG. Such tools are inexpensive, can help in earlier disease detection, and potentially improve prognosis.

## Plain Language Summary

The valves of the heart have flaps that open and close when the heart beats to maintain the flow of blood in the correct direction. Valvular disease, such as backflow or narrowing, puts additional strain upon heart muscles which can lead to heart failure. Usually, these conditions are diagnosed by doing an echocardiogram, an ultrasound scan of the heart and nearby blood vessels. The electrocardiogram (ECG) records the electrical signal generated by the heart and can be obtained more easily. We used deep learning neural networks, self-learning computer algorithms which excel at finding patterns within complex data. This enabled us to develop computer software able to diagnose valvular disease from ECGs. Earlier detection of such disease can help in improving overall outcome, while also reducing costs related to treatment.

## Abstract (web summary)

Vaid et al performed a multi-center retrospective cohort study using electrocardiograms from patients with mitral regurgitation to train a deep learning model to detect valvular disease and validated it in externally. They demonstrate the model could potentially enable earlier disease detection and improve overall prognosis.

## Introduction

Aortic Stenosis (AS) – obstruction to the flow of blood across the aortic valve secondary to reduction in valve aperture, and Mitral Regurgitation (MR) – retrograde flow of blood during systole across the mitral valve represent a growing health concern in developed nations. Taken together, valvular heart disease represents a substantial health burden affecting an estimated 2.5% of the general population^1^. Owing to the degenerative pathophysiology underlying chronic valvular disease, disease progression and prevalence are seen to vary in direct proportion with age^1^. Consequently, valvular disease has assumed central significance within cardiovascular medicine with significant attention being paid to early diagnosis and better management^2^. Approximately 20% of all cardiac surgeries are estimated to be for valvular repair^1^, and more recently, minimally invasive transcutaneous procedures have become more popular as first-line management^3,4^.

Diagnostic workflow for valvular disease is restricted to clinical workup^5,6^ due to no available biomarkers, or accepted guidelines for ECG interpretation. Physicians must manually auscultate patients for murmurs, and confirm their findings using echocardiography. Unfortunately, sensitivity for murmur auscultation is low^7,8^ and subject to significant inter-observer variability^9^. As a result, clinical suspicion tends to under-diagnose valvular disease by nearly 32%^1^. While echocardiography is accurate for diagnosis of valvular pathology, there remain considerable barriers in place towards its use as a screening modality in more resource constrained settings owing to concerns of logistics and trained personnel in sufficient numbers^10^.

The additive burdens of these factors foment a situation where there is a hidden burden of disease within the population subject to an overall worse prognosis following missed diagnosis, and disease progression^11^ in the absence of appropriate treatment^2^. This emphasizes the requirement of a widely available and inexpensive method to screen for, diagnose, or recommend intervention in patients with valvular disease.

The ECG is a powerful tool due to its low cost, availability, and applicability to a wide spectrum of diseases and has long been a mainstay of the cardiovascular diagnostic workflow. Despite these advantages, the ECG is limited by subjectivity in interpretation, and the requirement of diagnostic guidelines. Furthermore, human physicians cannot reliably appreciate minor ECG changes, leading to loss of valuable contextual and diagnostic information.

Machine learning is a mathematical approximation of human reasoning and intuition. Deep Learning (DL) is a subset of machine learning which utilizes neural networks to derive and attach semantic information to patterns within high dimensional data. Within the context of healthcare, and specifically ECG based diagnosis^12^, DL has been applied to diagnosis and risk prediction of arrythmias^13–15^, estimation of ventricular function^16,17^, predict risk of sudden cardiac death^18^, as well as detection of valvular disease^19–22^. While powerful in their own right, existing work on DL detection of valvular disease is limited by small sample sizes^19–21^, lack of statistical resampling^19–22^, lack of external validation^20,22^, and ethnically and racially homogenous cohorts^19–22^.

In this multi-center and externally validated study, we present DL algorithms which utilize ECG waveform data to detect valvular pathology from its effect on the electrical activity of the heart. Further, we consider the real-world implications and feasibility of such a model by assessing pre-echo diagnostic capability, and performance across a large cohort of socioeconomically and demographically diverse patient groups. Finally, we validate the assumption that machine learning can be used to guide clinical care by analyzing the rate of cardiac procedures with respect to model predictions. Our models achieved strong performance for diagnosis of Aortic Stenosis with an Area Under the Receiver Operating Characteristic Curve (AUROC) of 0.89 in internal testing, maintained in external validation at 0.81. For diagnosis of Moderate to Severe Mitral Regurgitation, our models achieved AUROC values of 0.88 and 0.81 in internal testing and external validation, respectively. In either case, performance was maintained in the presence of other left heart valvular pathology. On longitudinal follow up, patients diagnosed as true positives for Aortic Stenosis had a higher rate for minimally invasive valve replacement procedures.

## Methods

### Data source and patient population

Transthoracic Echocardiograms (echo) report processing, ECG preprocessing, and model selection are similar to methodologies described in Vaid et al.^17^. We utilized patient data from 2008–2020 from five New York City hospitals within the Mount Sinai Health System (MSHS). These hospitals, namely Mount Sinai Hospital, Mount Sinai Morningside, Mount Sinai Brooklyn, Mount Sinai West, and Mount Sinai Beth Israel serve a large and demographically diverse population (Tables 1 and 2).

**Table 1:** Population metrics: patients with Mitral Regurgitation (MR) in internal and external validation cohorts.

	Mitral Regurgitation^a^	Normal^b^	*p*	Mitral Regurgitation^a^	Normal^b^	*p*
	Internal testing cohort			External validation cohort		
Patients	21,740	94,872		1744	5116	0.51
Echo-ECG pairs	177,759	399,431		8919	21,320	1.4 ×10^−11^
Age	71.4 (71.3–71.5)	65.6 (65.5–65.6)	0	70.4 (70.1–70.7)	70.5 (70.3–70.7)	
Gender *n* (%)			0			
Male	10,763 (49.51)	50,886 (53.64)		873 (50.06)	2621 (51.23)	4.6 ×10^−18^
Female	10,977 (50.49)	43,986 (46.36)		871 (49.94)	2495 (48.77)	
Race *n* (%)			8.6 ×10^−244^			
American Indian	219 (1.01%)	1043 (1.1%)		–	–	
Asian	484 (2.23%)	2347 (2.47%)		–	21 (0.41%)	
Black	1958 (9.01%)	8257 (8.7%)		240 (13.76%)	784 (15.32%)	
Hispanic	1511 (6.95%)	6755 (7.12%)		21 (1.2%)	89 (1.74%)	
Other	2166 (9.96%)	9894 (10.43%)		544 (31.19%)	1498 (29.28%)	
Pacific Islander	27 (0.12%)	123 (0.13%)		–	–	
Unknown	8262 (38.0%)	41,318 (43.55%)		541 (31.02%)	1534 (29.98%)	2.5 ×10^−36^
White	7113 (32.72%)	25,135 (26.49%)		393 (22.53%)	1187 (23.2%)	5.6 ×10^−15^
Ventricular Rate	80.7 (80.5–81.0)	78.4 (78.3–78.5)	1.1 ×10^−145^	84.1 (83.2–85.0)	80.6 (80.0–81.1)	0.24
Atrial Rate	99.4 (98.5–100.3)	85.9 (85.6–86.2)	0	99.2 (96.3–102.1)	91.3 (89.8–92.7)	0.51
PR Interval	172.5 (172.0–173.0)	164.5 (164.3–164.7)	4.1 ×10^−24^	170.8 (169.0–172.6)	169.7 (168.7–170.8)	1.4 ×10^−11^
QTc Interval	467.5 (466.9–468.1)	448.4 (448.1–448.8)	0	473.2 (471.1–475.3)	458.3 (457.2–459.5)	9.3 ×10^−138^

*p*-values generated using ANOVA for continuous variables and Chi-square test for categorical variables.

^a^Mitral Regurgitation: Moderate to severe MR, Severe MR.

^b^Normal: No MR, Mild/Borderline/Trace MR, Moderate MR.

**Table 2:** Population metrics: patients with Aortic Stenosis (AS) in internal and external validation cohorts.

	Aortic Stenosis^a^	Normal^b^	*p*	Aortic Stenosis^a^	Normal^b^	*p*
Internal testing cohort			External validation cohort			
Patients	8883	111,681		516	7595	
Echo-ECG pairs	67,740	517,358		2360	29,880	
Age	78.4 (78.3–78.4)	65.4 (65.3–65.4)	0	78.9 (78.5–79.3)	70.4 (70.2–70.6)	5.6 ×10^−195^
Gender n (%)			3.3 ×10^−15^			0.11
Male	4610 (51.9%)	59,446 (53.23%)		283 (54.84)	3866 (50.9)	
Female	4273 (48.1%)	52,235 (46.77%)		233 (45.16)	3729 (49.1)	
Race *n* (%)			0			5.0 ×10^−24^
American Indian	68 (0.77%)	1192 (1.07%)		–	–	
Asian	136 (1.53%)	2755 (2.47%)		–	23 (0.3%)	
Black	431 (4.85%)	9807 (8.78%)		53 (10.27%)	1177 (15.5%)	
Hispanic	516 (5.81%)	7732 (6.92%)		–	128 (1.69%)	
Other	721 (8.12%)	11,927 (10.68%)		155 (30.04%)	2291 (30.16%)	
Pacific Islander	11 (0.12%)	140 (0.13%)		–	–	
Unknown	3551 (39.98%)	48,393 (43.33%)		141 (27.33%)	2282 (30.05%)	
White	3449 (38.83%)	29,735 (26.62%)		157 (30.43%)	1691 (22.26%)	
Ventricular Rate	76.6 (76.3–77.0)	78.8 (78.7–78.9)	0	78.9 (77.3–80.4)	80.8 (80.3–81.2)	7.2 ×10^−13^
Atrial Rate	91.8 (90.5–93.1)	86.9 (86.6–87.2)	0.056	87.2 (83.3–91.2)	89.7 (88.6–90.8)	0.26
PR Interval	177.9 (177.1–178.8)	164.5 (164.2 –164.8)	8.3 ×10^−99^	179.9 (176.3–183.5)	167.9 (167.1–168.7)	1.0 ×10^−88^
QTc Interval	460.3 (457.4–463.3)	449.4 (449.2–449.6)	8.6 ×10^−110^	465.2 (461.6–468.8)	455.8 (454.9–456.7)	2.03 ×10^−14^

*p*-values generated using ANOVA for continuous variables and Chi-square test for categorical variables.

^a^Aortic Stenosis: Moderate to severe AS, Severe AS.

^b^Normal: No AS, Mild/Borderline/Trace AS, Moderate AS.

**Fig. 1:**
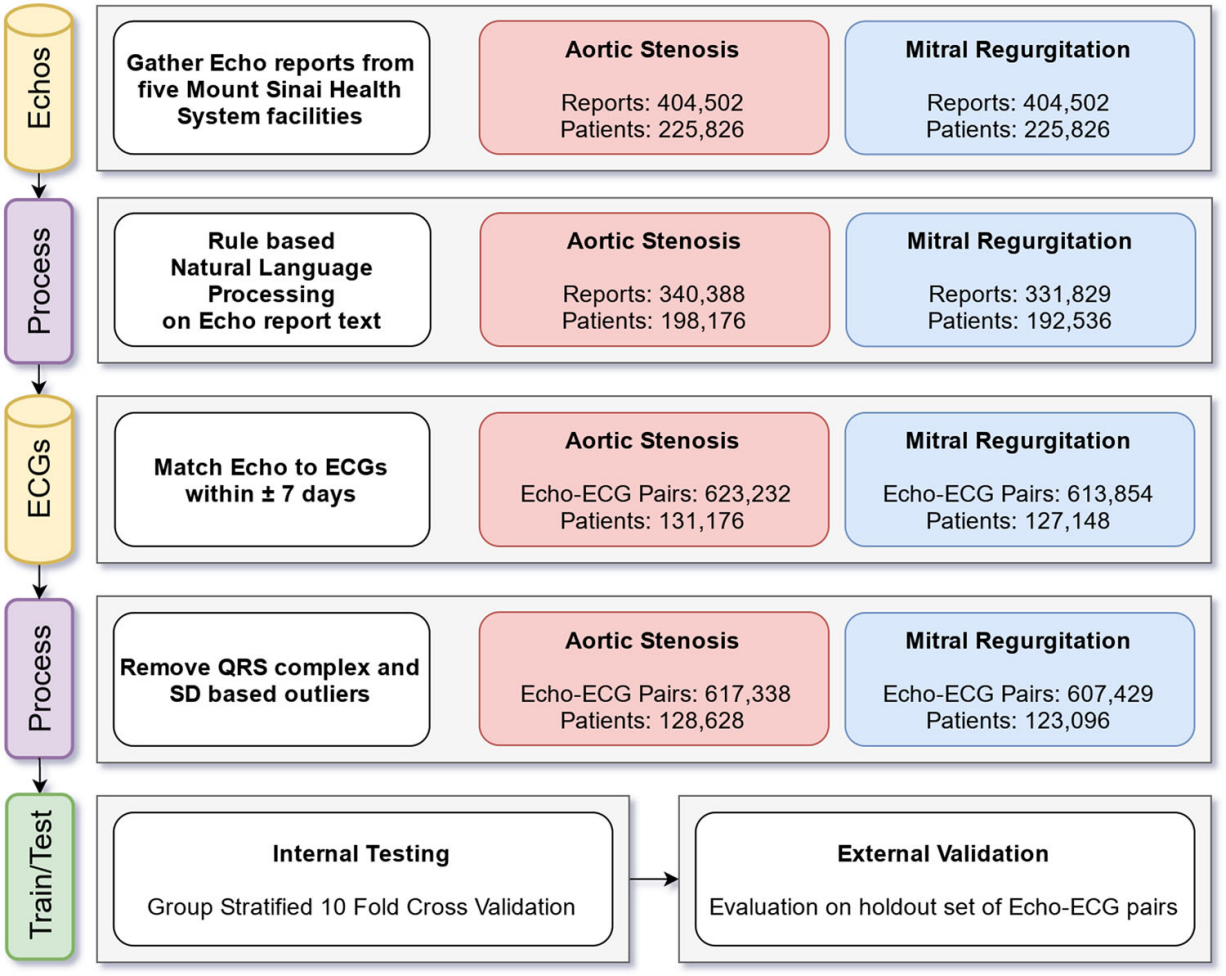
Flow diagram showing numbers of patients and paired ECG investigations at each step of data preprocessing. Numbers indicate investigations following initial data collection, followed by parsing echo reports for relevant diagnostic terms, temporal restriction, and removal of outliers based on mathematical analysis of waveforms.

We acquired pdf files which contained unstructured text corresponding to echo reports written by physicians. Collected reports contained the date of the investigation, and a unique Medical Record Number (MRN). ECG data were exported from the GE MUSE ECG system as structured .xml *(eXtensible Markup Language)* files containing MRN, date of the ECG, patient demographic details, ECG cart generated diagnoses, and raw waveform data. For each outcome as defined by an echo report we paired the echo derived label to any ECG performed within a period of ±7 days. This workflow is summarized in Fig. 1, and a flow diagram illustrating data extraction and analysis using a convolutional neural network is shown in Supplementary Fig. 1.

### Definition of outcomes

Status of either AS or MR was extracted from text of echo reports using Natural Language Processing (NLP)*.* An Echo-ECG pair was considered positive for the outcome in the presence of either moderate-to-severe; or severe valvular disease. Similarly, a pair was considered negative in the presence of either mild, borderline, or trace disease; moderate disease; or the valve being diagnosed as normal. Echo reports which did not comment on the status of either valve were excluded. Since there were only two possible outcomes, either task was considered an example of a binary classification problem.

### Data processing

#### Natural language processing

We developed a rule-based NLP approach to parse the unstructured text of each echo report. A set of rules was created iteratively by accounting for syntactic variation of text phrases within analyses of common parameters. In addition to AS/MR labels, these rules also extracted qualifiers regarding their severity. The final set of rules is enumerated in Supplementary Table 1. Annotated and anonymized samples of echo reports parsed using this method are provided in Supplementary Figs. 1, 2, and 3. Moderate-to-severe AS was designated as severe. While no official guideline exists for this delineation, we found comparable rates by cohort (14.9% of reports for internal testing and 21.9% for external validation) and across hospitals (12-30% of reports by facility) Annotated and anonymized samples of echo reports parsed using this method are provided in Supplementary Figs. 2, 3, and 4.

Performance of the NLP approach was evaluated by two faculty reviewers in a single-blind design. Each review contained 210 echo reports randomly sampled on NLP labeled normal, abnormal, and lack of reporting on valvular status.

#### ECG data

Waveform data is available within .xml files as a one-dimensional array of numbers or *vectors*. Each ECG is sampled at 500 Hz, and contains data for leads I, II, V1-V6. Vectors consist of either 5000 samples (10 s), or 2500 samples (5 s). To avoid potential imputation and padding related artifacts, each vector was attenuated to only the first 2500 samples. Data from leads III, aVF, aVL, and aVR was considered to provide no additional information since these leads can be derived from linear operations on the vectors of other leads^23^.

To aid removal of analog artefacts/recording errors^24^ present within the ECG, we utilized a median filter applied over a 2 s window, followed by a Butterworth Bandpass filter applied to the 0.5–40 Hz range. Further, to account for recordings with no lead information (flat line), or excess noise despite filter restrictions, we calculated the average QRS complex amplitude and the average standard deviation for each lead for the entire population. Any ECG with a lead average QRS complex amplitude, or an average standard deviation outside 2 standard deviations of the population mean for that value was excluded (Fig. 1).

The .xml files corresponding to ECGs which passed this quality control step were then parsed for demographics (patient age and sex) as well as certain cart extracted parameters embedded within the file (corrected QT interval, PR interval, Atrial rate, and Ventricular rate). The relationships between these extracted parameters are shown in the pairplots of Supplementary Figs. 5 and 6.

Waveforms corresponding to each ECG were plotted to an image. This image was combined with the tabular data extracted above as part of our modeling approach. Finally, no exclusions were performed based on recorded diagnoses associated with the ECG. We believe not having such exclusions would increase generalizability across pathologies.

### Model architecture

We utilized a combination neural network consisting of a Multi-Layer Perceptron (MLP) joined to an Efficientnet^25^ Convolution Neural Network (CNN) utilizing a joint fusion strategy^26^. Extracted (tabular) data was input into the MLP part of the neural network, while the image created from ECG data was converted into a tensor and input into the CNN part of the network. The final layer of the composite neural network consisted of one neuron and utilized *Sigmoid* activation. All models were trained using the *Binary Cross Entropy* loss function, and the *Adam* optimizer with a learning rate of 1e−4.

### Experimental design

We created an internal training and testing dataset using data collated from 4 MSHS facilities (Mount Sinai Hospital, Mount Sinai Brooklyn, Mount Sinai West, and Mount Sinai Beth Israel). All data collected from Mount Sinai Morningside was retained for a separate, unique external validation dataset. Data distribution across these datasets is detailed in Tables 1 and 2.

### Statistics and reproducibility

We utilized a Group Stratified K-fold cross validation design with K = 10. This sampling strategy prevents data leakage by treating each patient as a separate group, with each group being restricted to either the training or testing datasets. Stratified K-fold cross validation trains and tests model performance across K iterations, with each iteration’s training/testing splits keeping the same class distribution as the original dataset.

For the dual purposes of accounting for minor temporal variation between ECGs of the same patient, as well as improving model performance by way of data augmentation, each TTE report was paired to all ECGs performed within ±7 days of the echo during model training. During model evaluation, we emulated real-world deployment conditions by only considering the ECG closest to the TTE report within the 7-day time interval.

15% of testing data for each cross-fold iteration was utilized as an internal validation dataset. Following each epoch of training, performance was evaluated against this internal validation dataset. To prevent model overfitting, we implemented an early stopping approach to break the main training loop when performance on this internal validation dataset stopped increasing for 5 epochs. At this point, the model was evaluated on the remaining 85% of the internal testing data, and the external validation data.

Model performance was evaluated using threshold independent Area Under Receiver Operating Characteristic curve (AUROC), and Area Under Precision Recall Curve (AUPRC) metrics. Threshold dependent metrics such as Sensitivity, and Specificity were calculated based on an optimal threshold derived from the Youden J index^27^.

### Evaluation of performance in patient subgroups

Comorbidities such as systemic hypertension and a concomitant ipsilateral valvular lesion can affect both the diagnosis and development of valvular pathology. We extracted ICD diagnostic codes (ICD9: 401, 642*, 997.91; ICD10: I10, O10*, O13*, O16*) for patients close to the time of the ECG and performed an additional check of model performance in patients with either of isolated or concomitant valvular lesions.

### Validation of Aortic Stenosis model deployment

Transcatheter Aortic Valve Replacement (TAVR) is a minimally invasive procedure that obviates the need for conventional open-heart surgery, especially in patients with high surgical risk. We assessed the incidence of TAVRs with respect to model predictions.

Since TAVR is a relatively new procedure, we restricted our cohort to patients who had an ECG in 2015 or later. All echo reports with documented moderate-to-severe, or severe AS were labeled positive for the procedure, and model predictions for each cross-fold were labeled positive or negative based on the Youden index. Contingent on the ground truth of the echo derived label, predictions were labeled either true positive, true negative, false positive or false negative. For patients who had a TAVR, each prediction was then paired to the time of the procedure. Following this, we plotted cumulative incidence curves taking into account the time interval between each prediction:TAVR pair over a 5 year follow up period.

We also evaluated the development of valvular lesions in patients who were categorized as false positives over a 5-year follow-up period. For patients with repeat ECGs proximal to an echo, we fit a Kaplan–Meier model to the time of development of a valvular lesion from an initial false positive diagnosis.

### Comparison of performance with tabular models

We developed tabular XGBoost models to compare the performance of our DL approach to simpler methods which rely on ECG features. For diagnosis of either AS or MR for patients from the Internal testing cohort, we utilized machine extracted parameters as enumerated in Supplementary Table 5. Model performance was compared using the AUROC metric.

### Model interpretability

We utilized the captum framework for model interpretability owing to its integration with the PyTorch deep learning library, as well as support for multi-modal inputs. Plots were created showing the region of the plotted ECG contributing most to a prediction, in addition to showing the net contributions of both the waveform data and the extracted tabular data.

### Software and hardware

Data curation, processing and analysis was performed using the pandas, numpy, scikit-learn, PIL, torchvision, and PyTorch libraries within the Python programming language. NLP tasks were performed using the spaCy library. Code was run within custom docker containers created from official PyTorch docker images. Demographic performance plots and cumulative incidence curves were generated using the ggplot2 library within the R programming language. Models were trained on an Azure Cloud virtual machine on 4x NVIDIA v100 GPUs with 16GB VRAM each.

### IRB approval

This study was approved and informed consent was waived by the Icahn School of Medicine Institutional Review Board for utilization of retrospectively collected data (IRB-20-03271).

### Reporting summary

Further information on research design is available in the Nature Portfolio Reporting Summary linked to this article.

## Results

### Performance of rule-based NLP algorithm

We created a rule-based NLP approach to extract information about valvular status from the unstructured text contained within Echo reports. Performance at this task was evaluated by two faculty reviewers in a single-blind framework and quantified in terms of correctly classified labels, incorrectly classified labels, and missed labels. For this evaluation, valvular disease of any severity was considered as a demarcation between normal and abnormal.

From 420 outcomes in review for AS, we correctly classified 398, missed a label for 13, and incorrectly classified 1. For detected outcomes, this was an accuracy of 99.7%. For the 420 outcomes in review for MR, we correctly classified 379, missed labels on 37, and incorrectly classified 4. For detected outcomes, this was an accuracy of 99.1%. However, since all errors were towards detection of Borderline MR incorrectly detected as Normal, accuracy of input labels was assumed to not be affected. Overall performance is detailed in Supplementary Table 2.

### Performance at Mitral Regurgitation classification

We built a deep learning model to detect presence of moderate-to-severe, or severe MR. Data was collected for 607,429 Echo-ECG pairs for 123,096 patients and divided them into internal testing (7.11% prevalence) and external validation (7.32% prevalence) cohorts (Table 1). There was no correlation found between any extracted tabular variables (Supplementary Fig. 5).

**Fig. 2:**
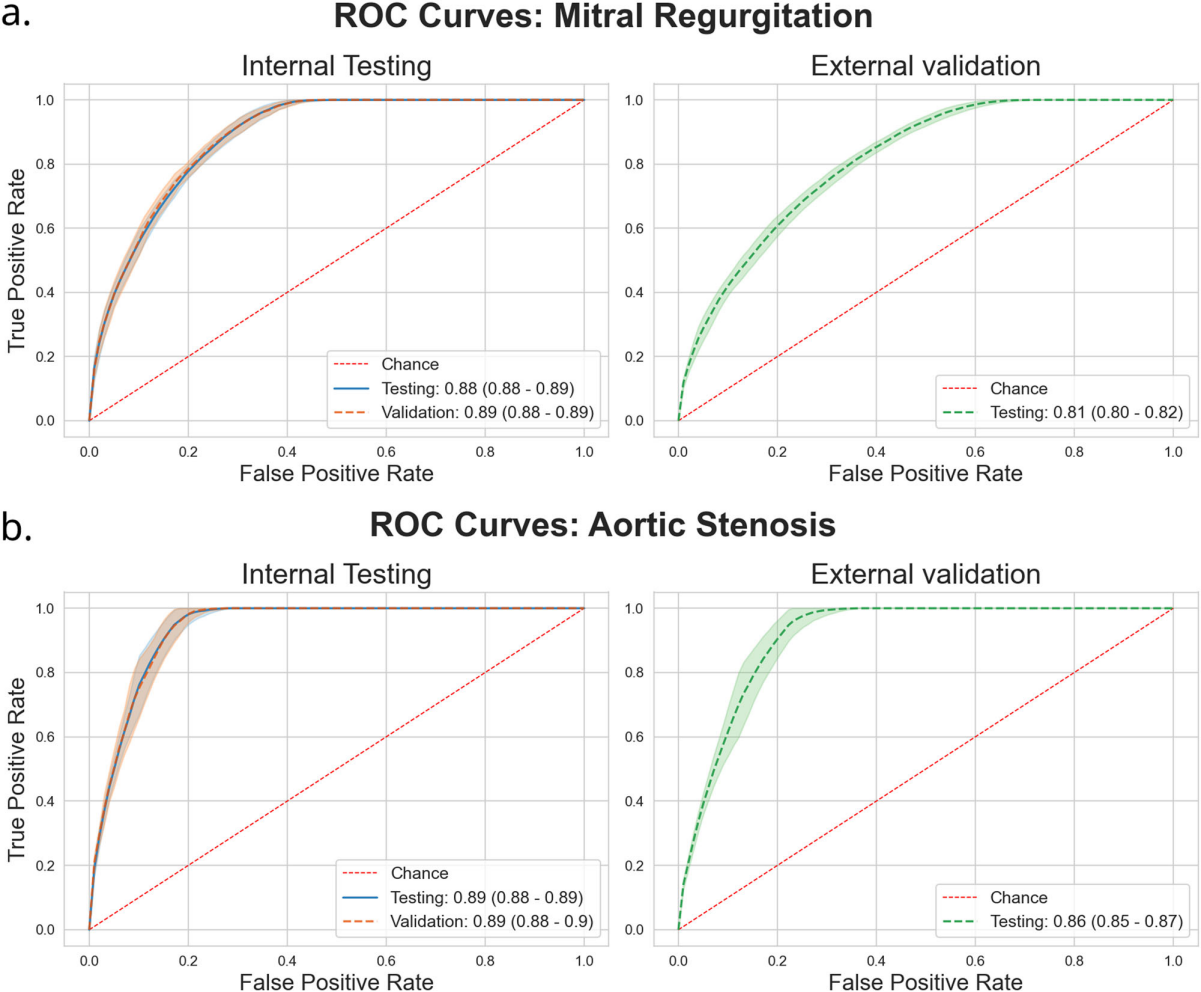
Receiver Operating Characteristic (ROC) Curves. Panel **a** Mitral Regurgitation. Panel **b** Aortic Stenosis. Area Under Curve (95% Confidence Interval) with shaded area around curve representing confidence interval. Red dashed line represents floor of performance as in the case of a hypothetical model making purely random predictions. Overall dataset size: 607,429 Echo-ECG pairs for 123,096 patients for Mitral Regurgitation. 617,338 Echo-ECG pairs for 128,628 patients for Aortic Stenosis.

**Fig. 3:**
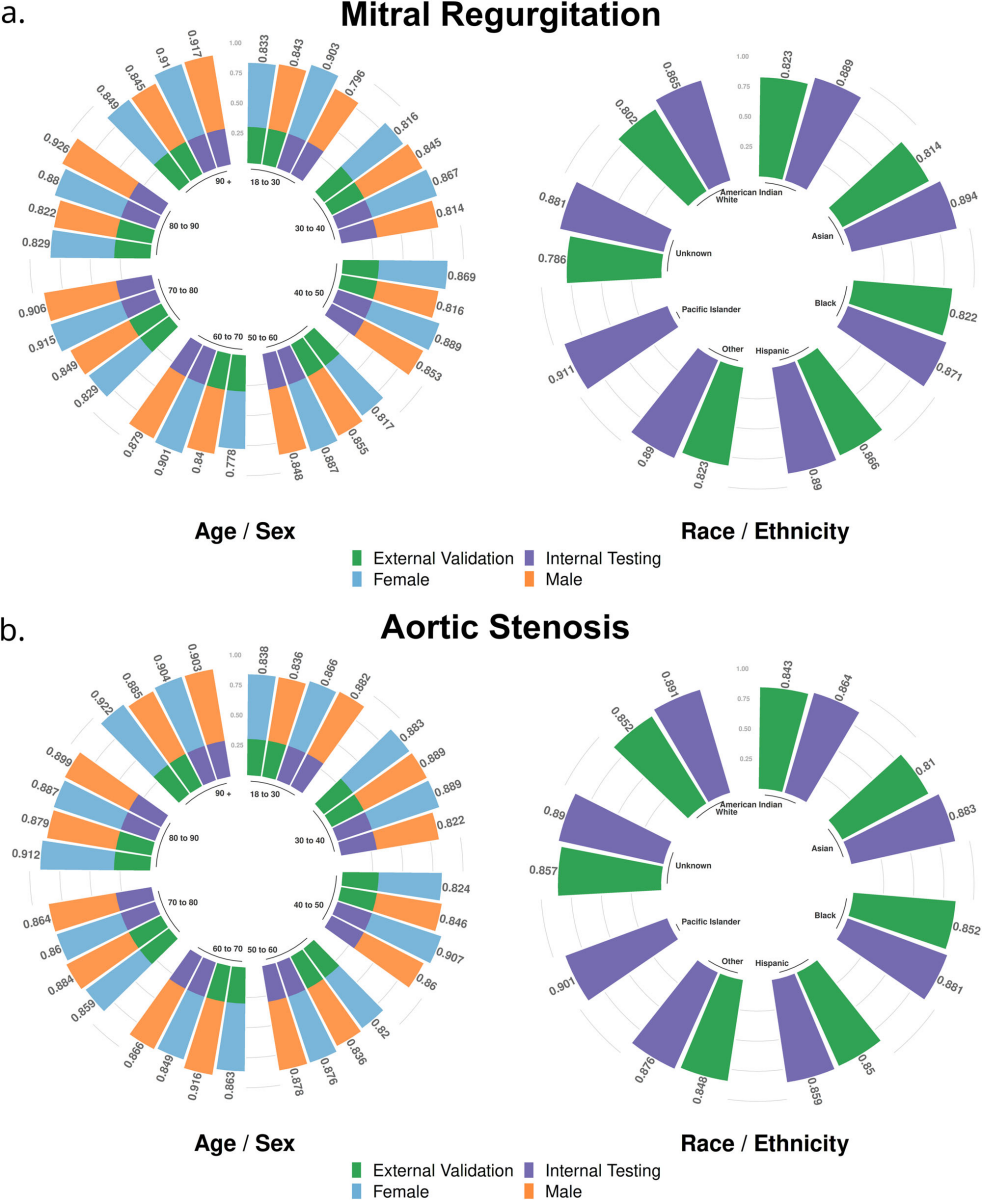
Model performance by age, sex, and race subgroups. Panel **a** Mitral Regurgitation. Panel **b** Aortic Stenosis. Values presented are Area Under Receiver Operating Characteristic Curve (AUROC). Bar segments at bottom delineate internal testing and external validation by color. Inner circles represent groups by age/US Census defined racial categories. *Source data for figure is available in* Supplementary Data 1–6.

**Fig. 4:**
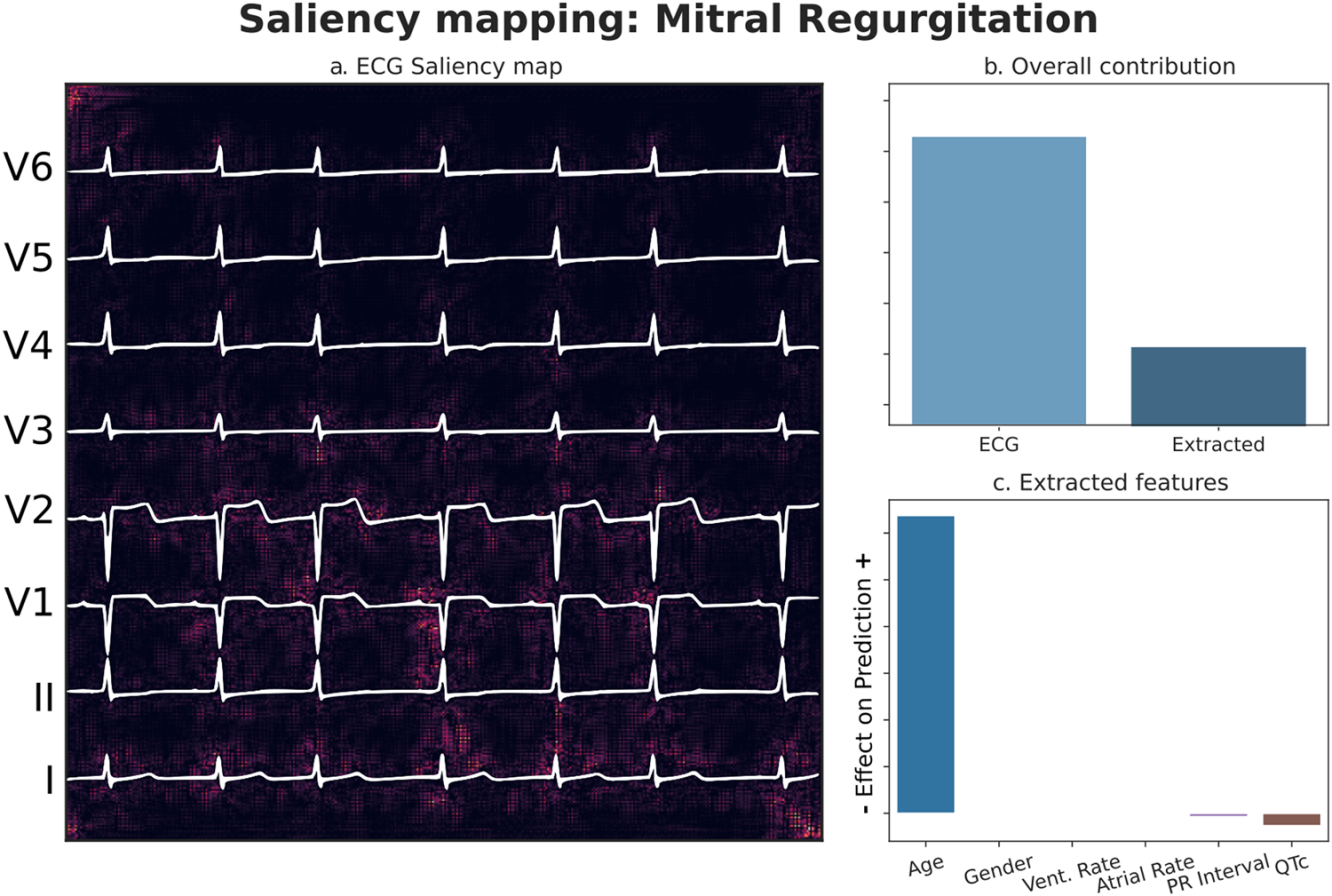
Model interpretability: Mitral Regurgitation. Panel **a** Input pixels most responsible for driving the prediction towards the outcome are highlighted. Panel **b** Relative contributions of waveform / tabular data to the final prediction. Panel **c** Relative importance of tabular features with respect to each other. *Patient (n* = *1) was positive for Mitral Regurgitation. Source data for figure is available in* Supplementary Data 7.

Model performance was strong in the internal testing dataset with an AUROC of 0.88 (95% CI: 0.88–0.89). This performance lowered to 0.81 (95% CI: 0.80–0.82) in the external validation dataset (Fig. 2). Interestingly, the AUPRC was 0.59 (95% CI: 0.57–0.61) in internal testing, but higher at 0.63 (95% CI: 0.61–0.65) in external validation (Supplementary Fig. 7). In either of internal testing and external validation, AUROC was seen to be constant across groups based on race, age, and sex (Fig. 3). Interpretability plots for MR classification highlighted QRS complexes as features that pushed the model towards a positive prediction as seen in Fig. 4. Overall model performance alongside threshold dependent metrics is summarized in Table 3.

**Table 3:** Classification performance.

Cohort	Internal testing	External validation
**Mitral regurgitation**		
% eval prevalence	17.40%	29.48%
AUROC	0.88 (0.88−0.89)	0.81 (0.8−0.82)
AUPRC	0.59 (0.57−0.61)	0.63 (0.61−0.65)
Sensitivity	0.94 (0.91−0.96)	0.83 (0.8−0.87)
Specificity	0.69 (0.66−0.71)	0.63 (0.58−0.67)
Positive Predictive Value	0.39 (0.37−0.40)	0.48 (0.47−0.50)
Negative Predictive Value	0.98 (0.97−0.99)	0.90 (0.89−0.92)
**Aortic Stenosis**		
% eval prevalence	7.11%	7.32%
AUROC	0.89 (0.88−0.89)	0.86 (0.85−0.87)
AUPRC	0.35 (0.34−0.37)	0.30 (0.28−0.31)
Sensitivity	0.93 (0.9−0.96)	0.92 (0.89−0.96)
Specificity	0.71 (0.69−0.74)	0.63 (0.6−0.66)
Positive Predictive Value	0.20 (0.18−0.22)	0.17 (0.15−0.18)
Negative Predictive Value	0.99 (0.99−1.00)	0.99 (0.99−0.99)

Youden J index used for calculation of Sensitivity, Specificity, Positive Predictive Value, and Negative Predictive value. *p*-values generated using ANOVA.

### Performance at Aortic Stenosis classification

We built a DL model to detect presence of moderate-to-severe, or severe AS. We accumulated data for 617,338 Echo-ECG pairs for 128,628 patients and divided them into internal testing (7.11% prevalence) and external validation (7.32% prevalence) cohorts (Table 2). Distribution of extracted ECG parameters was once again seen to have no correlation between any pair of variables (Supplementary Fig. 6).

Our model again achieved strong performance in internal testing with an AUROC of 0.89 (95% CI: 0.88–0.89). Performance was slightly lower in external validation at an AUROC of 0.86 (95% CI: 0.85–0.87) (Fig. 2). This trend was maintained with respect to AUPRC, with values of 0.35 (95% CI: 0.34–0.37) and 0.30 (95% CI: 0.28–0.31) in internal testing and external validation, respectively (Supplementary Fig. 8).

**Fig. 5:**
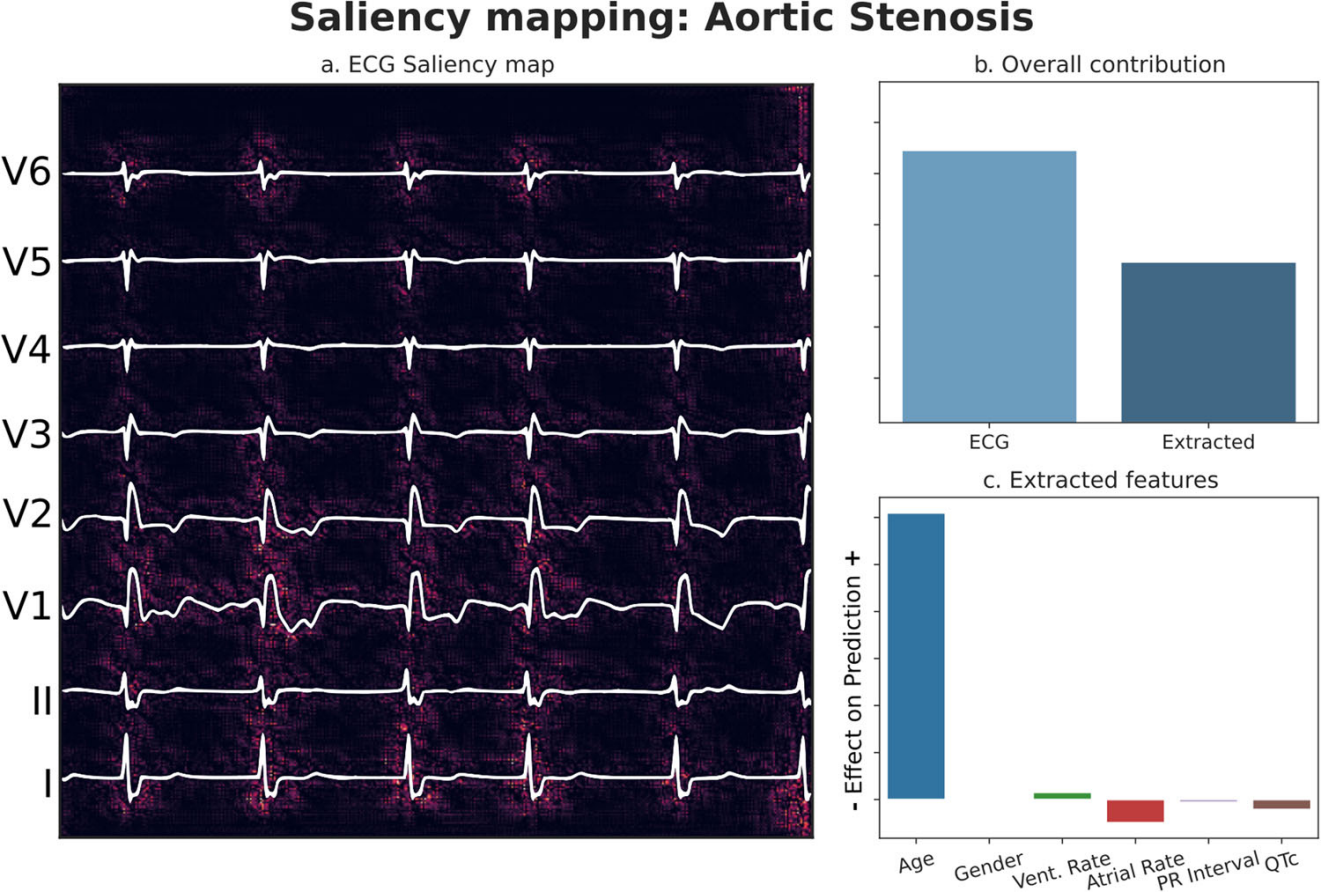
Model interpretability: Aortic Stenosis. Panel **a** Input pixels most responsible for driving the prediction towards the outcome are highlighted. Panel **b** Relative contributions of waveform / tabular data to the final prediction. Panel **c** Relative importance of tabular features with respect to each other. *Patient (n* = *1) was positive for Aortic Stenosis*. *Source data for figure is available in* Supplementary Data 7.

As seen for MR classification, model performance was roughly constant across demographic groups (Fig. 3), and interpretability plots highlighted QRS complexes driving the model towards prediction of the outcome (Fig. 5). Model performance was equivalent in both hypertensives and non-hypertensives in internal testing, and was slightly higher in hypertensives by an AUROC of 0.03 in external validation (Internal testing prevalence: 35%, External validation prevalence: 33.9%) (Supplementary Fig. 9).

Overall model performance alongside threshold dependent metrics is summarized in Table 3.

### Validation of Aortic Stenosis deployment using Transcatheter Aortic Valve Replacement incidence

**Fig. 6:**
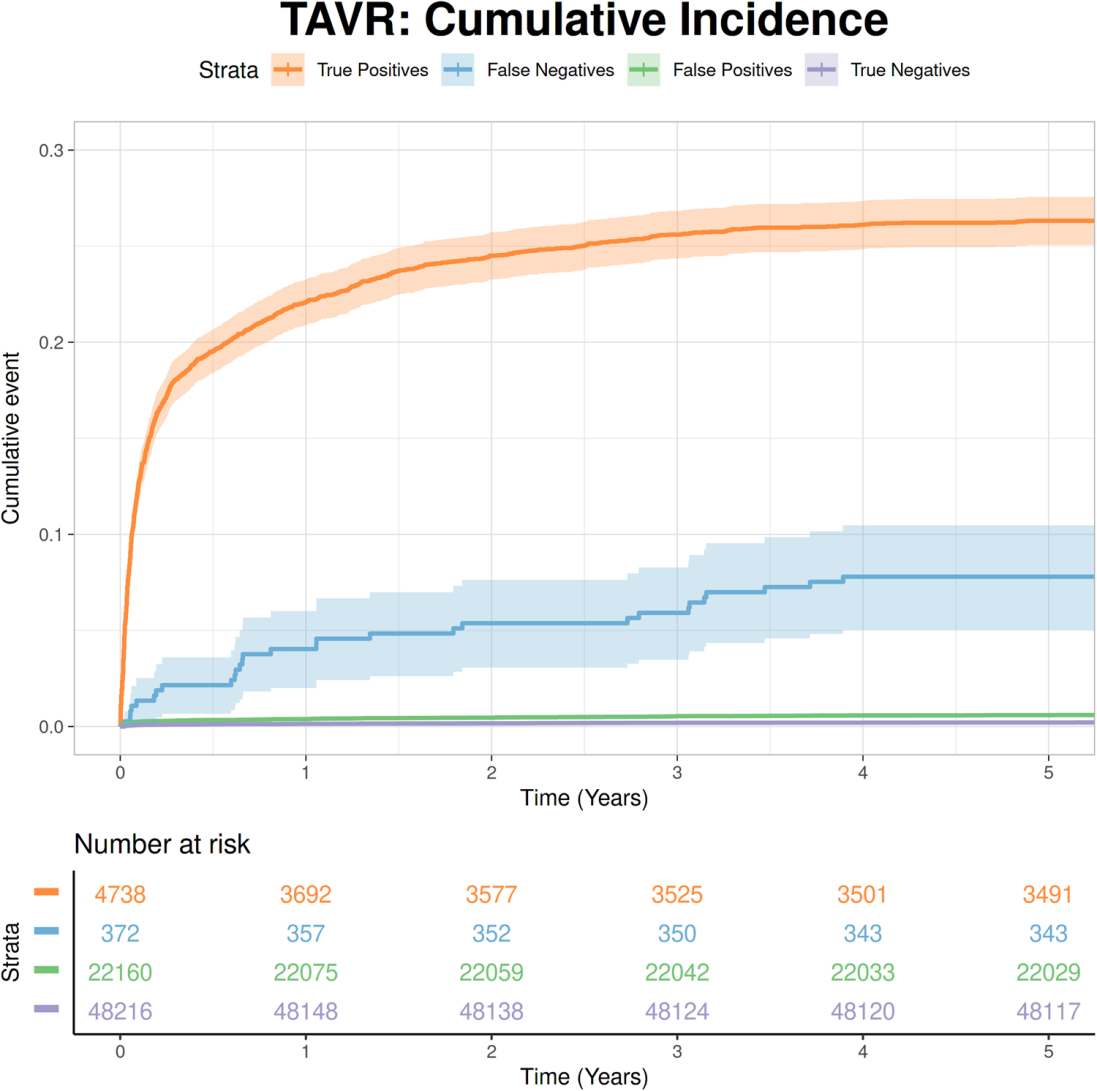
Cumulative incidence of Transcatheter Aortic Valve Replacement (TAVR) by model prediction. At risk numbers represent multiple predictions paired to each TAVR procedure for each patient prior to the date of the procedure. Shaded area around curve represents confidence interval. Follow up interval: 5 years.

We evaluated model performance with respect to each pair of TTE and TAVR incidence. We found that patients who were classified as true positives by the model had a much higher TAVR rate as opposed to those who were classified as false negatives or true negatives. Interestingly, we found that the TAVR rate was highest in true positives, followed by false negatives, false positives and finally true negatives (Fig. 6).

In addition, we found that the average time between a prediction:TAVR pair was much lower than other groups at 0.49 years in the true positive group. Total numbers of prediction pairs and unique procedures per group are summarized in Supplementary Table 3.

### Performance at pre-diagnostic echo Aortic Stenosis detection

We evaluated the performance of the AS classifier on ECGs collected in 4 different time intervals prior to the first diagnostic echo detailing the presence of moderate-to-severe, or severe AS. These time intervals were 3–6 months (2062 patients), 6–12 months (2096 patients) 12–18 months (2076 patients), and 18–24 months (2058 patients). For each time interval, all ECGs for each patient were considered, and cases were balanced against an equivalent number of controls.

**Fig. 7:**
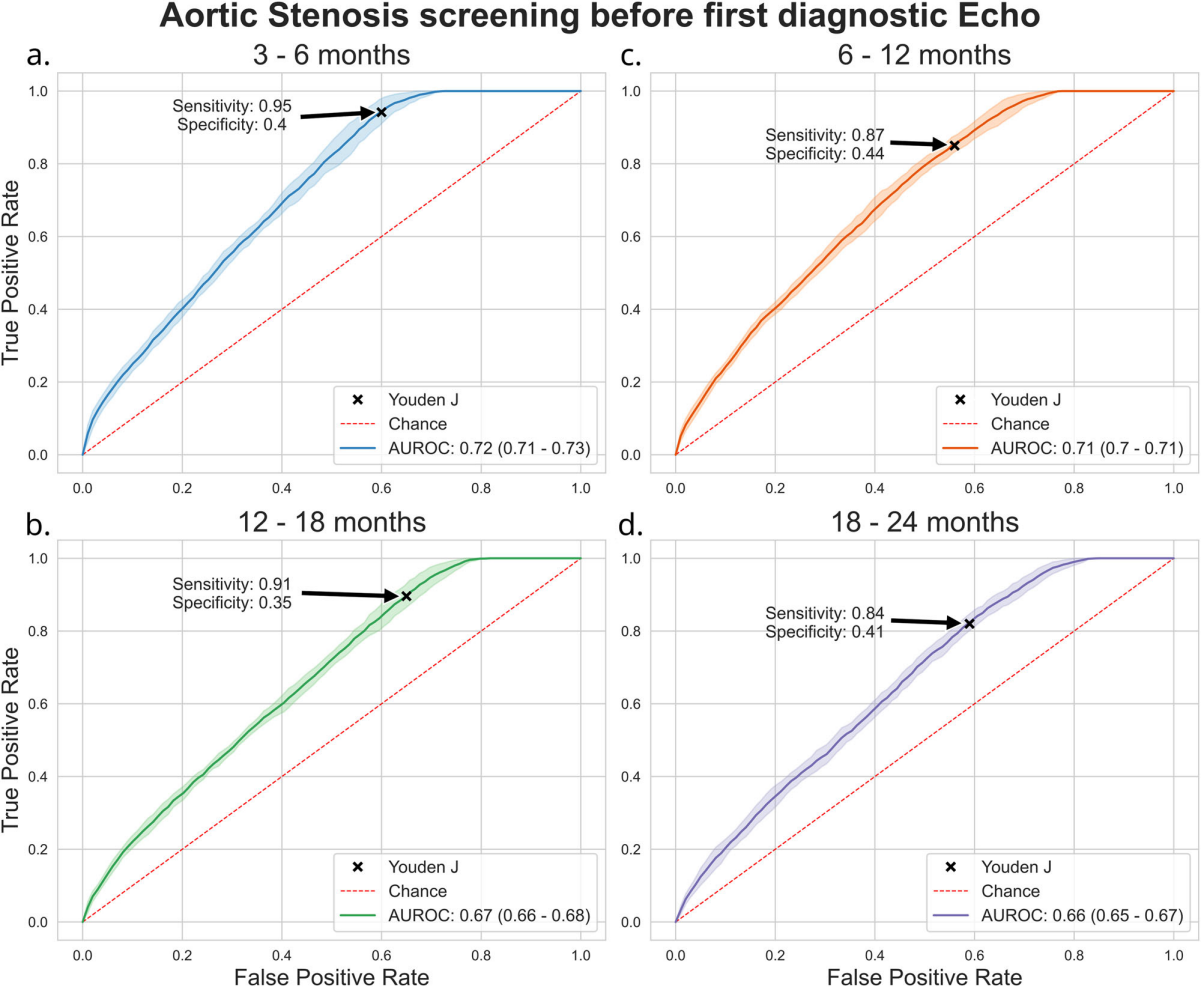
Model performance at detection of Aortic Stenosis prior to diagnostic echo. Shaded area around curve represents confidence interval. Highlighted points on each curve demonstrate optimal sensitivity and specificity as derived by the Youden J. Panel **a** *n* = 2062 patients (3–6 months), Panel **c** 2096 patients (6–12 months), Panel **b** 2076 patients (12–18 months) and Panel **d** 2058 patients (18–24 months) *AUROC: Area Under Receiver Operating Characteristic Curve.*

Model performance was seen to increase in inverse proportion to the time interval between the ECG and the diagnostic echo. At 18–24 months, AUROC was 0.66. This increased to 0.67 at 12–18 months, 0.72 at 6–12 months and 3–6 months. By using the Youden index to derive an optimal threshold, our model achieved sensitivity scores of 0.84, 0.91, 0.87, and 0.95 over the same time intervals (Fig. 7, Supplementary Table 4).

### Performance in patients with concomitant left heart valvular lesions

We evaluated the performance of both classifiers with respect to performance in concomitant AS + MR, and compared this to performance for isolated valvular lesions. We found that for AS classification, AUROC was higher for patients with concomitant AS + MR in both internal testing (AS + MR: 0.91; Isolated AS: 0.86) and external validation (AS + MR: 0.89; Isolated AS: 0.85).

Similarly, the MR classifier also performed better for patients with concomitant AS + MR – with an improvement in AUROC of 0.07 for both internal testing (AS + MR: 0.92; Isolated MR: 0.85) and external validation (AS + MR: 0.88; Isolated MR: 0.81) (Supplementary Fig. 10).

### Valvular lesion development in false positive predictions

We considered model predictions over a 5-year follow up period for all patients for whom we had more than one ECG:Echo pair, and fit Kaplan–Meier curves to the time of first diagnosis of both Aortic Stenosis and Mitral Regurgitation. We found that the cumulative incidence of the corresponding valvular lesion was higher for patients who had an initial False Positive prediction (AS: 4.5% vs 1.7%; MR: 24.8% vs 10.1%), over those who had an initial True Negative prediction (Supplementary Fig. 11).

## Discussion

AS and MR represent a hidden health burden within the population that is amenable to more inexpensive and widely available screening tools. We collected around 600,000 ECGs for a socioeconomically and demographically diverse group of 120,000 New York City patients who were administered care within multiple centers of the Mount Sinai Health System. We created an accurate NLP pipeline capable of extraction of labels from the unstructured text of echo reports. Utilizing these labels, we developed and evaluated deep learning models capable of detecting moderate-to-severe, or severe AS/MR. We analyzed performance of either model across racial/ethnic, age, and sex-based groups. For AS, we performed a longitudinal analysis of model performance by considering model performance at detection prior to a diagnostic echo, as well as validating model predictions against Transcatheter Aortic Valve Replacement procedures.

Deep learning (DL) represents a powerful set of tools capable of discerning patterns within complex data. While DL requires more computational resources and data points than traditional methods using tabular data, we found that use of DL achieved better results than traditional machine learning models. Further, it does not require manual feature selection, or expert input into feature selection. This is invaluable for problems where human expertise cannot isolate specific markers or patterns of disease.

DL models must be tested before deployment, and external validation is a necessary requirement towards making a final assessment of model quality in terms of generalizability. Biases assisting model performance within an internal dataset – especially one taken from a single facility may not be replicated within an external cohort. This may severely compromise the validity and performance of any real-world implementation of a model^20^. We were encouraged to see our models had little change in performance in going from internal testing to external validation. Similarly, racial biases inform not only availability of healthcare, but also compliance with treatment. In addition, certain disease processes are more prevalent and more severe in certain racial groups. Prior work has been limited to East Asian^19–21^ and Caucasian^22^ populations. Our training population is representative of the racial and socioeconomic diversity of New York City, and we found that our models performed consistently across each racial, age, and sex-based group.

Additionally, prior work does not include resampling. A single training-testing data split cannot ensure equitable distribution of easy or hard to predict cases within training and testing data. Consequently, reported model performance may be erroneously elevated. By performing Group Stratified Cross validation, we ensure we capture the variability inherent to each split of data.

AS is a chronic, progressive condition – often taking months or years to increase in severity to where it becomes overtly symptomatic or warrants clinical suspicion. Following from this, we validated model performance within a longitudinal framework by considering a scenario where such a model could be used as a screening tool. In calculating performance at detecting AS before a patient had a diagnostic echo within the ambit of normal clinical workflow, we found that our model’s performance increased monotonically up to the time of the diagnostic echo. This pattern suggests the model is capable of tracking progression of ECG changes indicative of AS.

We also evaluated how our model’s predictions agreed with physician decisions about whom to consider for a TAVR procedure. We found that both the absolute number of procedures, as well as the procedure rate was much higher in patients who had true positive predictions as opposed to false negative predictions. Furthermore, even patients who had false positive predictions had an overall higher rate of TAVR procedures than true negatives. We posit that this trend shows such patients have a higher risk of developing symptomatic AS eventually requiring intervention. Interestingly, the rate of TAVR procedures was greater in true positives in comparison to false negatives. We surmise this follows from the application of the Youden J Index. Due to the tradeoff between sensitivity and specificity^28^, lower risk positives (with a correspondingly lower procedure rate) as classified as negatives.

Overall, we believe this makes a strong case for our model’s ability to gauge disease severity. Fine-tuning such a model^29^ on labels derived from whether or not a patient had a TAVR may help guide clinical decisions regarding the requirement of or suitability for the procedure and will form the basis of future work.

Our work is better understood in light of certain limitations. We paired echos to ECGs within a ± 7-day period. Severe MR can develop acutely secondary to ischemic and non-ischemic pathologies affecting the heart. This may decrease model accuracy owing to the myocardium not having enough time to get acclimated. This could be a reason for model performance dropping within internal testing and external validation cohorts for MR, and further evidences the importance of external validation prior to model deployment. Additional random error may have been introduced by the labels generated by the NLP pipeline, despite high accuracy upon manual review (>99%). Low outcome prevalence in the study population led to proportionally low Positive Predictive Values (PPV) for both outcomes. While this limitation cannot be easily resolved without additional data, we were encouraged to find our models’ performance exceed prior work^19,21,22^ at this metric. External validation was performed at the Mount Sinai Morningside hospital, which while part of the same health system as the hospitals from which training data was collected, serves a different patient population. There was no patient overlap for this external validation site. We found that the internal and external validation cohorts had a significantly different distribution of demographic and extracted ECG parameters (Table 1). However, further external validation from another health system is warranted in future work.

We have incorporated the capabilities of DL into successfully deriving additional information from inexpensive, widely available ECGs for outcomes that do not have an established set of diagnostic guidelines. Such models can be used to screen patients or direct them along appropriate care pathways.

## Supplementary information

Supplementary Data 1

Supplementary Data 2

Supplementary Data 3

Supplementary Data 4

Supplementary Data 5

Supplementary Data 6

Supplementary Data 7

Peer Review File

Description of Additional Supplementary Files

Reporting Summary

Supplementary Material

## Data Availability

The raw data is not publicly available because it contains privileged and protected patient information. Further detail is available on request from the corresponding author. The source data for Fig. 3 is available in Supplementary Data 1–6. The source data for Fig. 4 is available in Supplementary Data 7.

## Code availability

Program code detailing the neural network pipeline is available at https://github.com/akhilvaid/LeftHeartValvularDisease and is linked at https://zenodo.org/record/6375847^30^
