# Supplementary material for: Multi-center retrospective cohort study applying deep learning to electrocardiograms to identify left heart valvular dysfunction
Source: Commun Med (Lond). 2023 Feb 14;3:24. doi: 10.1038/s43856-023-00240-w (PMC9929085; doi:10.1038/s43856-023-00240-w)
Supplement: Supplementary file 9 — Description of Additional Supplementary Files [file 43856_2023_240_MOESM9_ESM.pdf]

Description of Additional Supplementary Files

File Name: Supplementary Data 1

Description: Figure 3 Aortic Stenosis Performance by Age

File Name: Supplementary Data 2

Description: Figure 3 Aortic Stenosis Performance by Race

File Name: Supplementary Data 3

Description: Figure 3 Aortic Stenosis Indicator Stumps

File Name: Supplementary Data 4

Description: Figure 3 Mitral Regurgitation Performance by Age

File Name: Supplementary Data 5

Description: Figure 3 Mitral Regurgitation Performance by Race

File Name: Supplementary Data 6

Description: Figure 3 Mitral Regurgitation Indicator Stumps

File Name: Supplementary Data 7

Description: Supplementary data for Figure 4 and 5 barplots.
