# Supplementary material for: Multi-center retrospective cohort study applying deep learning to electrocardiograms to identify left heart valvular dysfunction
Source: Commun Med (Lond). 2023 Feb 14;3:24. doi: 10.1038/s43856-023-00240-w (PMC9929085; doi:10.1038/s43856-023-00240-w)
Supplement: Supplementary file 11 — Supplementary Material [file 43856_2023_240_MOESM11_ESM.pdf]

Supplementary Figures

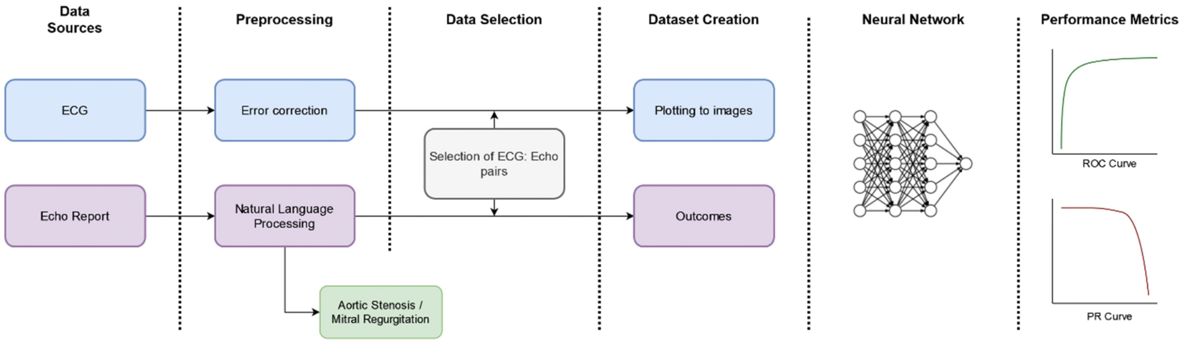

Supplementary Figure 1: Study Flow diagram

Data is collected as ECGs and echo reports. Preprocessing involves removal of outliers, and correction of baseline recording errors for ECGs. For echo reports, preprocessing involves rule based natural language processing to detect diagnostic terms. Data from these sources are paired, and analyzed using a neural network, followed by generation of performance metrics.

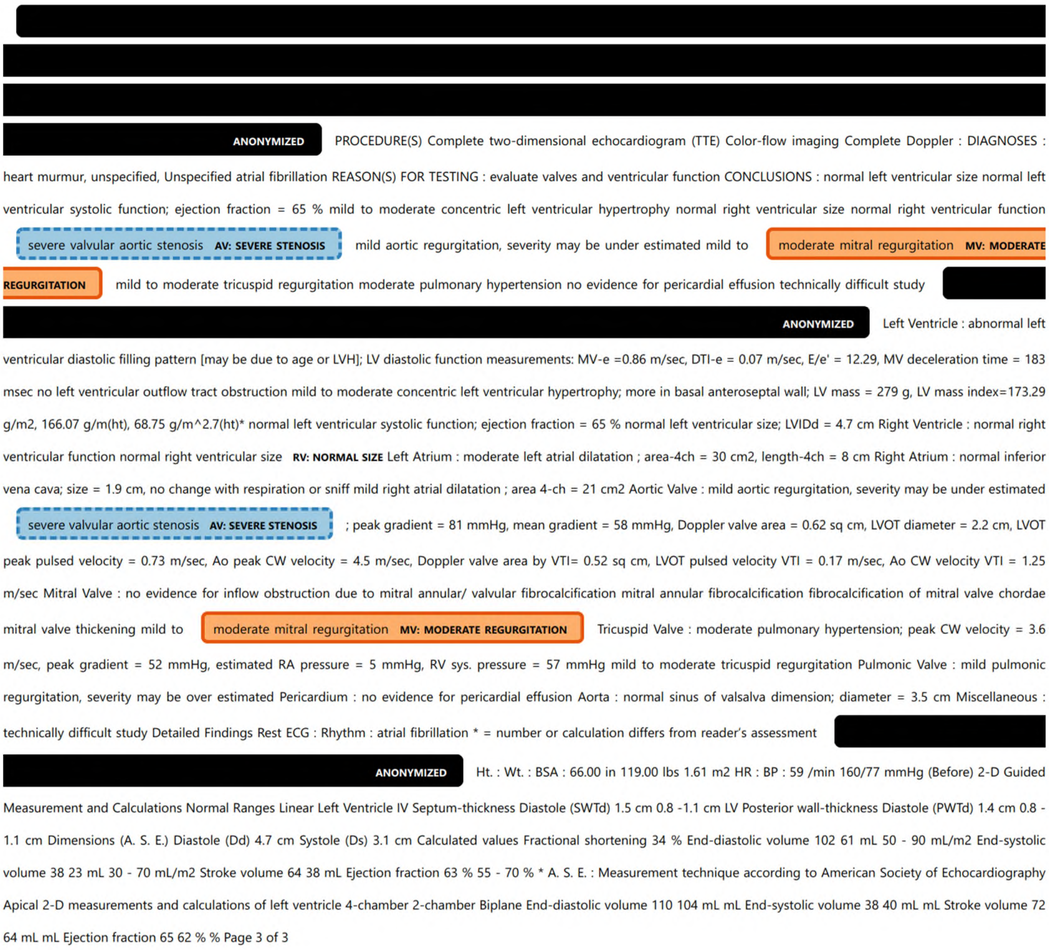

[redacted]

[redacted] ANONYMIZED PROCEDURE(S) Complete two-dimensional echocardiogram (TTE) Color-flow imaging Complete Doppler : DIAGNOSES : heart murmur, unspecified, Unspecified atrial fibrillation REASON(S) FOR TESTING : evaluate valves and ventricular function CONCLUSIONS : normal left ventricular size normal left ventricular systolic function; ejection fraction = 65 % mild to moderate concentric left ventricular hypertrophy normal right ventricular size normal right ventricular function severe valvular aortic stenosis **AV: SEVERE STENOSIS** mild aortic regurgitation, severity may be under estimated mild to moderate mitral regurgitation **MV: MODERATE REGURGITATION** mild to moderate tricuspid regurgitation moderate pulmonary hypertension no evidence for pericardial effusion technically difficult study [redacted] ANONYMIZED Left Ventricle : abnormal left ventricular diastolic filling pattern [may be due to age or LVH]; LV diastolic function measurements: MV-e =0.86 m/sec, DTI-e = 0.07 m/sec, E/e' = 12.29, MV deceleration time = 183 msec no left ventricular outflow tract obstruction mild to moderate concentric left ventricular hypertrophy; more in basal anteroseptal wall; LV mass = 279 g, LV mass index=173.29 g/m2, 166.07 g/m(ht), 68.75 g/m^2.7(ht)* normal left ventricular systolic function; ejection fraction = 65 % normal left ventricular size; LVIDd = 4.7 cm Right Ventricle : normal right ventricular function normal right ventricular size **RV: NORMAL SIZE** Left Atrium : moderate left atrial dilatation ; area-4ch = 30 cm2, length-4ch = 8 cm Right Atrium : normal inferior vena cava; size = 1.9 cm, no change with respiration or sniff mild right atrial dilatation ; area 4-ch = 21 cm2 Aortic Valve : mild aortic regurgitation, severity may be under estimated severe valvular aortic stenosis **AV: SEVERE STENOSIS** ; peak gradient = 81 mmHg, mean gradient = 58 mmHg, Doppler valve area = 0.62 sq cm, LVOT diameter = 2.2 cm, LVOT peak pulsed velocity = 0.73 m/sec, Ao peak CW velocity = 4.5 m/sec, Doppler valve area by VTI= 0.52 sq cm, LVOT pulsed velocity VTI = 0.17 m/sec, Ao CW velocity VTI = 1.25 m/sec Mitral Valve : no evidence for inflow obstruction due to mitral annular/ valvular fibrocalcification mitral annular fibrocalcification fibrocalcification of mitral valve chordae mitral valve thickening mild to moderate mitral regurgitation **MV: MODERATE REGURGITATION** Tricuspid Valve : moderate pulmonary hypertension; peak CW velocity = 3.6 m/sec, peak gradient = 52 mmHg, estimated RA pressure = 5 mmHg, RV sys. pressure = 57 mmHg mild to moderate tricuspid regurgitation Pulmonic Valve : mild pulmonic regurgitation, severity may be over estimated Pericardium : no evidence for pericardial effusion Aorta : normal sinus of valsalva dimension; diameter = 3.5 cm Miscellaneous : technically difficult study Detailed Findings Rest ECG : Rhythm : atrial fibrillation * = number or calculation differs from reader's assessment [redacted] ANONYMIZED Ht. : Wt. : BSA : 66.00 in 119.00 lbs 1.61 m2 HR : BP : 59 /min 160/77 mmHg (Before) 2-D Guided Measurement and Calculations Normal Ranges Linear Left Ventricle IV Septum-thickness Diastole (SWTd) 1.5 cm 0.8 -1.1 cm LV Posterior wall-thickness Diastole (PWTd) 1.4 cm 0.8 - 1.1 cm Dimensions (A. S. E.) Diastole (Dd) 4.7 cm Systole (Ds) 3.1 cm Calculated values Fractional shortening 34 % End-diastolic volume 102 61 mL 50 - 90 mL/m2 End-systolic volume 38 23 mL 30 - 70 mL/m2 Stroke volume 64 38 mL Ejection fraction 63 % 55 - 70 % * A. S. E. : Measurement technique according to American Society of Echocardiography Apical 2-D measurements and calculations of left ventricle 4-chamber 2-chamber Biplane End-diastolic volume 110 104 mL mL End-systolic volume 38 40 mL mL Stroke volume 72 64 mL mL Ejection fraction 65 62 % % Page 3 of 3

Supplementary Figure 2: Sample annotated NLP processed echo report

Each highlighted section illustrates a diagnosis detected by the natural language processing algorithm. Blue: AV Stenosis. Orange: Mitral Regurgitation

[redacted] ANONYMIZED PROCEDURE(S) Complete two-dimensional echocardiogram (TTE) Color-flow imaging Complete Doppler : DIAGNOSES : s/p mitral valve replacement REASON(S) FOR TESTING : evaluate valves and ventricular function CONCLUSIONS : Abnormal Mitral Tissue Prosthesis: partially obstructed and high gradient; cannot exclude fibrocalcification, partial thrombosis or vegetation ; suggest TEE normal left ventricular size moderate concentric left ventricular hypertrophy overall moderate decreased left ventricular systolic function (segmental); ejection fraction = 28 % LV function varies with R-R interval mild left atrial dilatation minimal mitral regurgitation **MV: BORDERLINE REGURGITATION** mild to moderate tricuspid regurgitation moderate pulmonary hypertension study performed with patient supine mild to moderate decreased right ventricular function mild right ventricular dilatation technically difficult study Definity® precision microbubble contrast used to enhance endocardial border definition [redacted] ANONYMIZED Left Ventricle : LV function varies with R-R interval moderate concentric left ventricular hypertrophy; LV mass = 271 g, LV mass index=167.28 g/m2, 169.38 g/m(ht), 76.18 g/m^2.7(ht)* overall moderate decreased left ventricular systolic function (segmental); severe hypocontractility of basal anteroseptal, mid anteroseptal, apical septal, apical walls; mild hypocontractility of mid anterior, apical anterior walls; ejection fraction = 28 %* normal left ventricular size; LVIDd = 4.6 cm Right Ventricle : mild to moderate decreased right ventricular function mild right ventricular dilatation right ventricle not well seen. Left Atrium : mild left atrial dilatation ; area-4ch = 21 cm2, length-4ch = 7 cm Right Atrium : normal inferior vena cava; size = 1.4 cm normal right atrial size; area 4-ch = 18 cm2 Aortic Valve : aortic sclerosis probable minimal aortic regurgitation no evidence for valvular aortic stenosis **AV: NO STENOSIS** Mitral Valve : Abnormal Mitral Tissue Prosthesis: partially obstructed and high gradient; cannot exclude fibrocalcification, partial thrombosis or vegetation ; suggest TEE consistent with abnormal mitral prosthesis (tissue); pressure half-time = 180 millisec, mean MV gradient = 14 mmHg, peak gradient = 28 mmHg moderate mitral stenosis; pressure half-time = 180 millisec, area by Doppler = 1.22 cm2 minimal mitral regurgitation **MV: BORDERLINE REGURGITATION** Tricuspid Valve : moderate pulmonary hypertension; peak CW velocity = 3.7 m/sec, peak gradient = 55 mmHg, estimated RA pressure = 5 mmHg, RV sys. pressure = 60 mmHg mild to moderate tricuspid regurgitation Pulmonic Valve : mild pulmonic regurgitation Pericardium : no evidence for pericardial effusion Aorta : normal sinus of valsalva dimension; diameter = 2.6 cm Miscellaneous : study performed with patient supine [redacted] ANONYMIZED Definity® precision microbubble contrast used to enhance endocardial border definition * = number or calculation differs from reader's assessment [redacted] ANONYMIZED Ht. : Wt. : BSA : 63.00 in 132.00 lbs 1.62 m2 HR : BP : 53 /min 138/67 mmHg (Before) 2-D Guided Measurement and Calculations Normal Ranges Linear Left Ventricle IV Septum-thickness Diastole (SWTd) 1.5 cm 0.8 -1.1 cm LV Posterior wall-thickness Diastole (PWTd) 1.4 cm 0.8 - 1.1 cm Dimensions (A. S. E.) Diastole (Dd) 4.6 cm Systole (Ds) 3.6 cm Calculated values Fractional shortening 22 % End-diastolic volume 97 61 mL 50 - 90 mL/m2 End-systolic volume 54 34 mL 30 - 70 mL/m2 Stroke volume 43 27 mL Ejection fraction 44 % 55 - 70 % * A. S. E. : Measurement technique according to American Society of Echocardiography Apical 2-D measurements and calculations of left ventricle 4-chamber 2-chamber Biplane End-diastolic volume 83 mL End-systolic volume 60 mL Stroke volume 23 mL Ejection fraction 28 % Page 4 of 4

Supplementary Figure 3: Sample annotated NLP processed echo report

Each highlighted section illustrates a diagnosis detected by the natural language processing algorithm.

Blue: AV Stenosis. Orange: Mitral Regurgitation

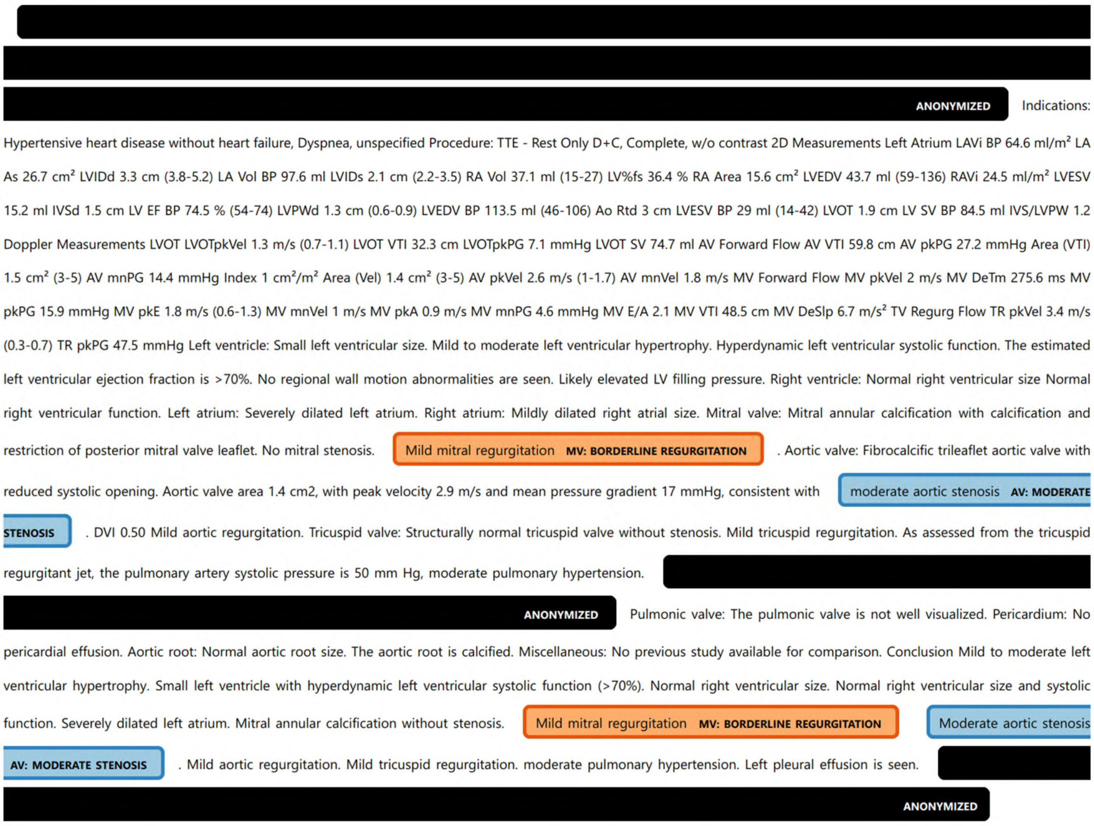

[redacted] ANONYMIZED Indications: Hypertensive heart disease without heart failure, Dyspnea, unspecified Procedure: TTE - Rest Only D+C, Complete, w/o contrast 2D Measurements Left Atrium LAVi BP 64.6 ml/m^2 LA As 26.7 cm^2 LVIDd 3.3 cm (3.8-5.2) LA Vol BP 97.6 ml LVIDs 2.1 cm (2.2-3.5) RA Vol 37.1 ml (15-27) LV%fs 36.4 % RA Area 15.6 cm^2 LVEDV 43.7 ml (59-136) RAVi 24.5 ml/m^2 LVESV 15.2 ml IVSd 1.5 cm LV EF BP 74.5 % (54-74) LVPWd 1.3 cm (0.6-0.9) LVEDV BP 113.5 ml (46-106) Ao Rtd 3 cm LVESV BP 29 ml (14-42) LVOT 1.9 cm LV SV BP 84.5 ml IVS/LVPW 1.2 Doppler Measurements LVOT LVOTpkVel 1.3 m/s (0.7-1.1) LVOT VTI 32.3 cm LVOTpkPG 7.1 mmHg LVOT SV 74.7 ml AV Forward Flow AV VTI 59.8 cm AV pkPG 27.2 mmHg Area (VTI) 1.5 cm^2 (3-5) AV mnPG 14.4 mmHg Index 1 cm^2/m^2 Area (Vel) 1.4 cm^2 (3-5) AV pkVel 2.6 m/s (1-1.7) AV mnVel 1.8 m/s MV Forward Flow MV pkVel 2 m/s MV DeTm 275.6 ms MV pkPG 15.9 mmHg MV pkE 1.8 m/s (0.6-1.3) MV mnVel 1 m/s MV pkA 0.9 m/s MV mnPG 4.6 mmHg MV E/A 2.1 MV VTI 48.5 cm MV DeSlp 6.7 m/s^2 TV Regurg Flow TR pkVel 3.4 m/s (0.3-0.7) TR pkPG 47.5 mmHg Left ventricle: Small left ventricular size. Mild to moderate left ventricular hypertrophy. Hyperdynamic left ventricular systolic function. The estimated left ventricular ejection fraction is >70%. No regional wall motion abnormalities are seen. Likely elevated LV filling pressure. Right ventricle: Normal right ventricular size Normal right ventricular function. Left atrium: Severely dilated left atrium. Right atrium: Mildly dilated right atrial size. Mitral valve: Mitral annular calcification with calcification and restriction of posterior mitral valve leaflet. No mitral stenosis. Mild mitral regurgitation MV: BORDERLINE REGURGITATION . Aortic valve: Fibrocalcific trileaflet aortic valve with reduced systolic opening. Aortic valve area 1.4 cm2, with peak velocity 2.9 m/s and mean pressure gradient 17 mmHg, consistent with moderate aortic stenosis AV: MODERATE STENOSIS . DVI 0.50 Mild aortic regurgitation. Tricuspid valve: Structurally normal tricuspid valve without stenosis. Mild tricuspid regurgitation. As assessed from the tricuspid regurgitant jet, the pulmonary artery systolic pressure is 50 mm Hg, moderate pulmonary hypertension. [redacted] ANONYMIZED Pulmonic valve: The pulmonic valve is not well visualized. Pericardium: No pericardial effusion. Aortic root: Normal aortic root size. The aortic root is calcified. Miscellaneous: No previous study available for comparison. Conclusion Mild to moderate left ventricular hypertrophy. Small left ventricle with hyperdynamic left ventricular systolic function (>70%). Normal right ventricular size. Normal right ventricular size and systolic function. Severely dilated left atrium. Mitral annular calcification without stenosis. Mild mitral regurgitation MV: BORDERLINE REGURGITATION Moderate aortic stenosis AV: MODERATE STENOSIS . Mild aortic regurgitation. Mild tricuspid regurgitation. moderate pulmonary hypertension. Left pleural effusion is seen. [redacted] ANONYMIZED

Supplementary Figure 4: Sample annotated NLP processed echo report

Each highlighted section illustrates a diagnosis detected by the natural language processing algorithm.

Blue: AV Stenosis. Orange: Mitral Regurgitation

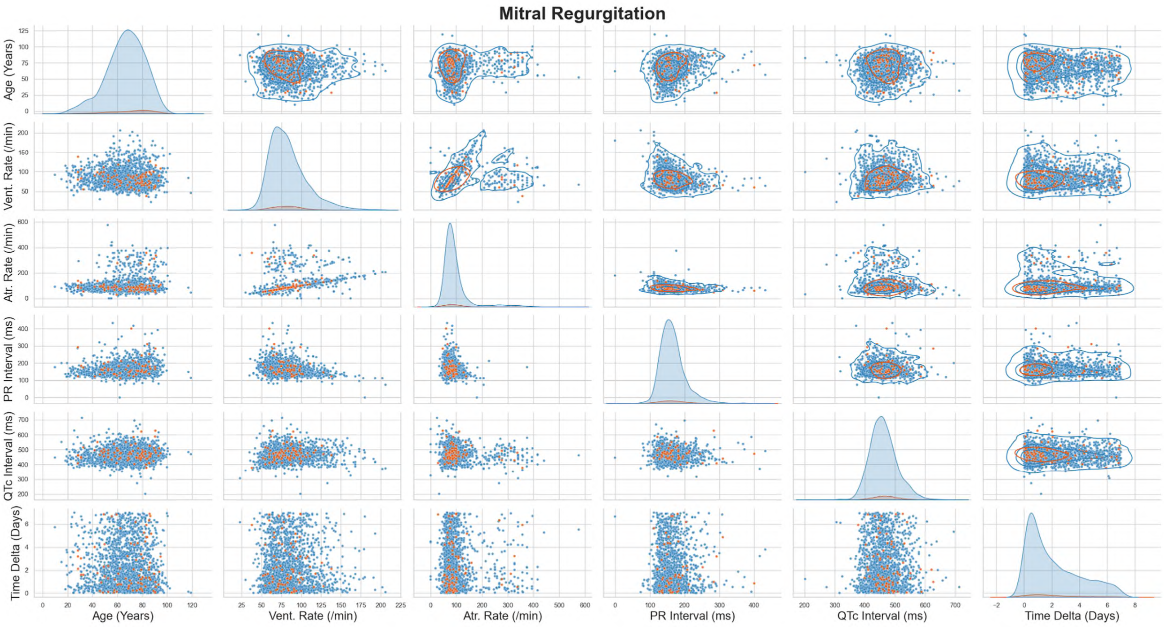

Supplementary Figure 5: Mitral Regurgitation: Pairplot showing relationships between extracted variables. Patient populations randomly sampled to n=2000 to prevent overplotting.

Orange: Moderate to severe MR, Severe MR

Blue: No MR, Mild/Borderline/Trace MR, Moderate MR

Lines: Kernel density estimates

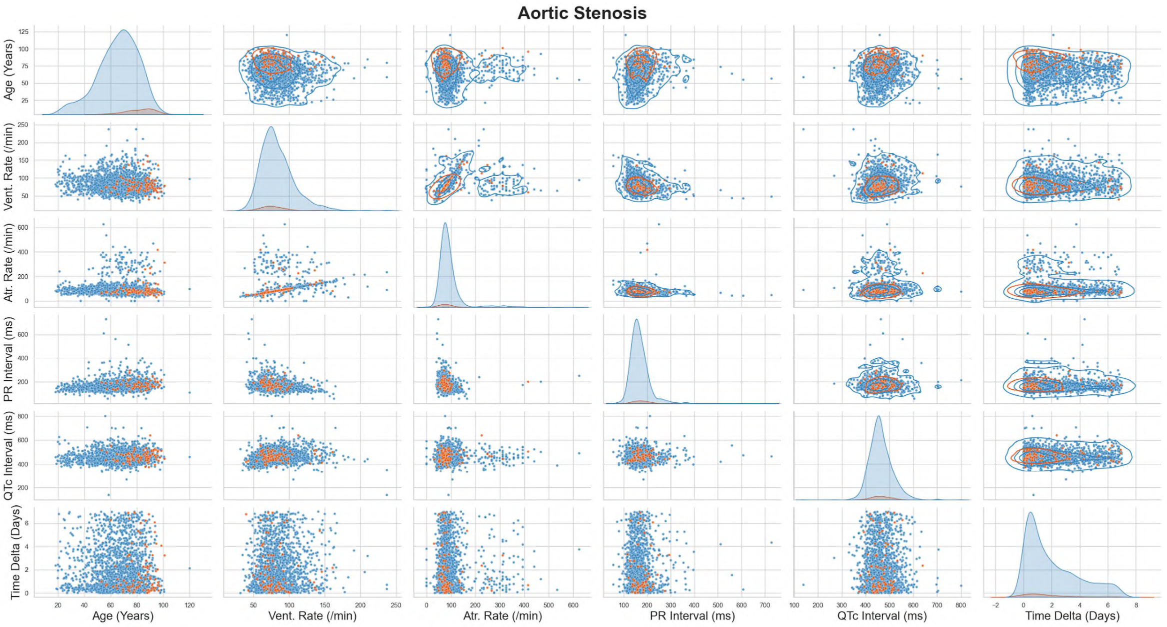

Supplementary Figure 6: Aortic Stenosis: Pairplot showing relationships between extracted variables. Patient populations randomly sampled to n=2000 to prevent overplotting.

Orange: Moderate to severe AS, Severe AS

Blue: No AS, Mild/Borderline/Trace AS, Moderate AS

Lines: Kernel density estimates

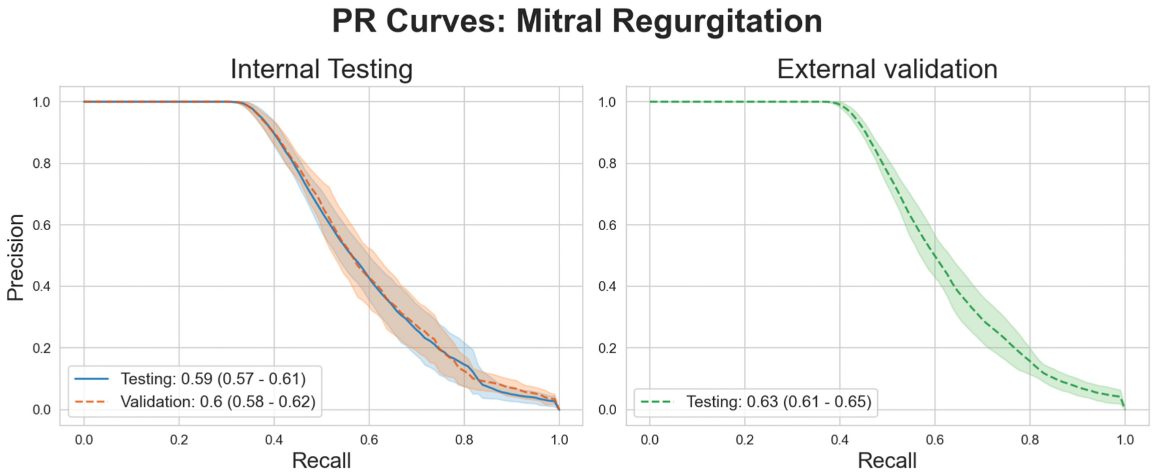

Supplementary Figure 7: Mitral Regurgitation: Precision Recall curves
Legend shows Area Under the Precision Recall Curve (AUPRC). Shaded area around curve represents confidence interval.

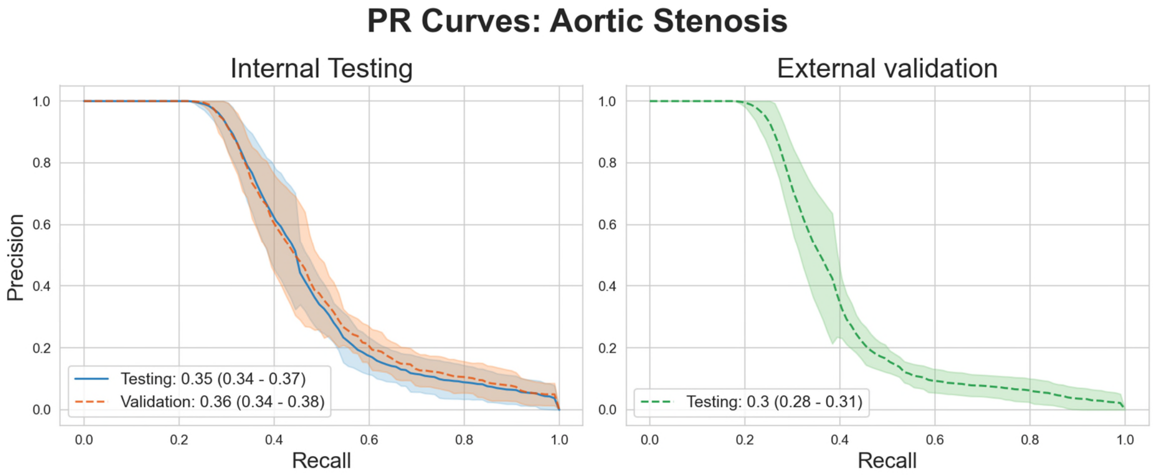

Supplementary Figure 8: Aortic Stenosis: Precision Recall curves.
Legend shows Area Under the Precision Recall Curve (AUPRC). Shaded area around curve represents confidence interval.

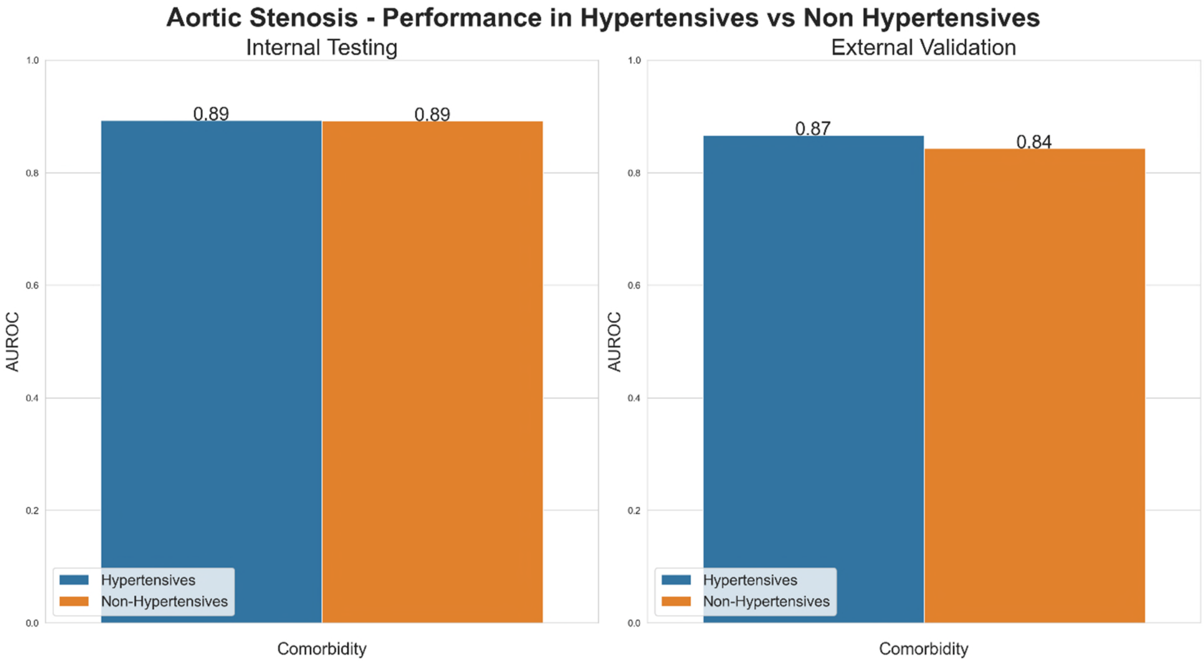

Supplementary Figure 9: Aortic Stenosis: Classifier performance variation with hypertension Hypertension can precipitate changes within the myocardium similar to aortic stenosis. This plot illustrates difference in performance in patients with diagnosed hypertension as opposed to those without.

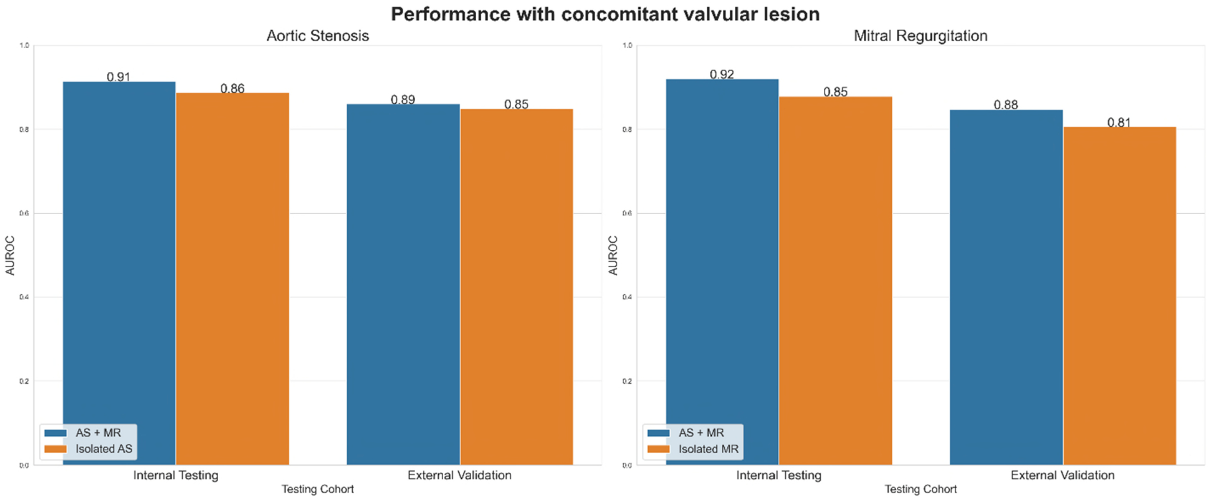

Supplementary Figure 10: Classifier performance in concomitant Aortic Stenosis + Mitral Regurgitation compared to isolated valvular lesion. Valvular lesions may occur together and serve to confound model performance. This plot highlights differences in performance in patients with either of, or both valvular lesions.

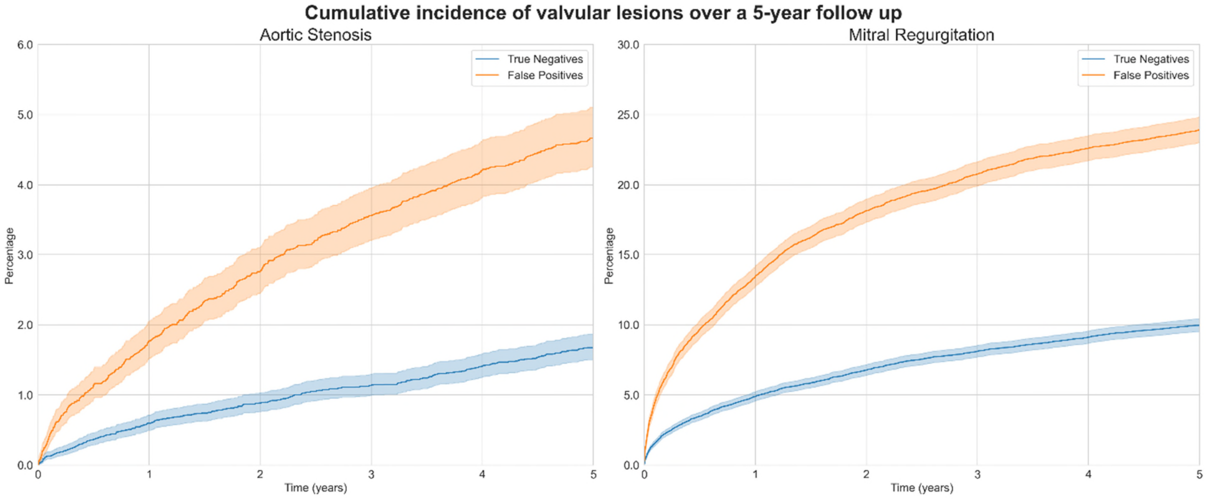

Supplementary Figure 11: Cumulative incidence of valvular lesion in model predicted false positives vs true negatives over a 5-year follow-up period.
Shaded area around curve shows confidence interval.

Supplementary Tables

Rule	Example Positive
**No Aortic Stenosis**	
normal {*} aortic valve	Normal trileaflet aortic valve
no aortic {*} stenosis	No aortic valvular stenosis
aortic valve {*} {*} without stenosis	Aortic valve is trileaflet without stenosis
no evidence {*} aortic {*} stenosis	No evidence of aortic valve stenosis
**Aortic Stenosis**	
{trace | minimal | mild} aortic {*} stenosis	Trace aortic valvular stenosis
moderate aortic {*} stenosis	Moderate aortic valve stenosis
moderate {*} severe aortic {*} stenosis	Moderate to severe aortic valve stenosis
severe aortic {*} stenosis	Severe aortic valve stenosis
**No Mitral Regurgitation**	
normal {*} mitral valve	Normal mitral valve
no mitral {*} {regurgitation | insufficiency}	No mitral valvular regurgitation

<table>
<tr><td></td><td></td></tr>
<tr><td colspan="2">Mitral Regurgitation</td></tr>
<tr><td>{trace | minimal | mild} mitral {*} {regurgitation | insufficiency}</td><td>Trace mitral valvular regurgitation</td></tr>
<tr><td>moderate mitral {*} {regurgitation | insufficiency}</td><td>Moderate mitral valvular regurgitation</td></tr>
<tr><td>moderate {*} severe mitral {*} {regurgitation | insufficiency}</td><td>Moderate to severe mitral valvular regurgitation</td></tr>
<tr><td>severe mitral {*} {regurgitation | insufficiency}</td><td>Severe mitral valvular regurgitation</td></tr>
</table>

Supplementary Table 1: NLP Rules

Natural Language Processing rules and example positives. Curly brackets imply choices or special processing

** : wildcard - one or no words*

| : Either of these words

**Outcome**	**Normal***			**Abnormal***			**No mention in report**		
	✓	✗	Total	✓	✗	Total	✓	✗	Total
**Reviewer I**									
AS	99	1	109	68	0	69	32	0	32
MR	17	0	23	144	3	157	30	0	30
**Reviewer II**									
AS	120	0	128	47	0	49	32	0	33
MR	17	0	21	140	2	162	32	0	33

Supplementary Table 2: NLP performance

Outcomes were sampled in equal proportion from NLP detected *Normal / Abnormal / No mention in report*. Therefore, result distribution is not representative of population distribution. The *Total* column includes outcomes missed by the NLP algorithm. Such reports and any matched ECGs were excluded. Errors in MR detection were all for Borderline MR detection.

AS: Aortic Stenosis, MR: Mitral Regurgitation

**Normal/Abnormal: Outcome of any severity included in abnormal.*

Prediction	Prediction:TAVR pairs	Total (unique) procedures in group	Average time between prediction and procedure
True Positives	4738	1248	0.49 years
False Negatives	372	29	1.20 years
False Positives	22160	133	1.04 years
True Negatives	48126	102	1.43 years

Supplementary Table 3: Model validation against TAVR procedures. Youden J index used for classification of prediction probability into positive and negative.

Time interval before diagnostic echo	Patients	Area Under Receiver Operating Characteristic Curve
3-6 months	2062	0.720
6-12 months	2096	0.715
12-18 months	2076	0.669
18-24 months	2058	0.659

Supplementary Table 4: Model performance at detection of moderate-to-severe, or severe Aortic Stenosis within different time intervals prior to the first diagnostic echo.

Features	AUROC
**Mitral Regurgitation**	
PR Interval, QT Interval, QRS duration, R Wave amplitude, T wave amplitude, J point location, Associated Diagnoses: Right Bundle Branch Block, Left Ventricular Hypertrophy, Right Axis deviation, Left Axis Deviation	0.68 (0.67 - 0.69)
Age, Gender, PR Interval, QT Interval, QRS duration, Ventricular Rate	0.67 (0.65 - 0.68)
**Aortic Stenosis**	
PR Interval, QT Interval, QRS duration, R Wave amplitude, T wave amplitude, J point location, Associated Diagnoses: Right Bundle Branch Block, Left Ventricular Hypertrophy, Right Axis deviation, Left Axis Deviation	0.69 (0.68 - 0.7)
Age, Gender, PR Interval, QT Interval, QRS duration, Ventricular Rate	0.78 (0.76 - 0.8)

Supplementary Table 5: Performance comparison with XGBoost model using tabular data in internal testing cohort.
