# Supplementary material for: Multi-center retrospective cohort study applying deep learning to electrocardiograms to identify left heart valvular dysfunction
Source: Commun Med (Lond). 2023 Feb 14;3:24. doi: 10.1038/s43856-023-00240-w (PMC9929085; doi:10.1038/s43856-023-00240-w)
Supplement: Supplementary file 8 — Peer Review File [file 43856_2023_240_MOESM8_ESM.pdf]

nature portfolio

Peer Review File

Deep learning on electrocardiograms to derive left heart valvular dysfunction: A multi-center retrospective cohort study with external validation

Open Access This file is licensed under a Creative Commons Attribution 4.0 International License, which permits use, sharing, adaptation, distribution and reproduction in any medium or format, as long as you give appropriate credit to the original author(s) and the source, provide a link to the Creative Commons license, and indicate if changes were made. In the cases where the authors are anonymous, such as is the case for the reports of anonymous peer reviewers, author attribution should be to 'Anonymous Referee' followed by a clear attribution to the source work. The images or other third party material in this file are included in the article's Creative Commons license, unless indicated otherwise in a credit line to the material. If material is not included in the article's Creative Commons license and your intended use is not permitted by statutory regulation or exceeds the permitted use, you will need to obtain permission directly from the copyright holder. To view a copy of this license, visit http://creativecommons.org/licenses/by/4.0/.

This manuscript has been previously reviewed at another Nature Portfolio journal. This document only contains reviewer comments and rebuttal letters for versions considered at Communications Medicine.

REVIEWERS' COMMENTS:

Reviewer #1 (Remarks to the Author): expertise in ECG

The manuscript is significantly improved after review. I have no further questions

[ED: this reviewer also commented favourably on your response to Reviewer #2s comments.]

Reviewer #3 (Remarks to the Author): expertise in AI

The authors addressed the concerns I raised earlier through additional experiments and descriptions in the text. Two small additional comments:
1) The acronym "TTE" should be defined before its first use.
2)Can the authors release the model? If not, then providing a specific reason for why not is important. The model's availability could help future researchers build off of the work, and as the authors describe this is an important future step. If the model is unable to be released due to privacy concerns, how can it be used in other institutions or cohorts?
